# Web-Based Service Provision of HIV, Viral Hepatitis, and Sexually Transmitted Infection Prevention, Testing, Linkage, and Treatment for Key Populations: Systematic Review and Meta-analysis

**DOI:** 10.2196/40150

**Published:** 2022-12-22

**Authors:** Ping Teresa Yeh, Caitlin Elizabeth Kennedy, Ayako Minamitani, Rachel Baggaley, Purvi Shah, Annette Verster, Niklas Luhmann, Maeve Brito de Mello, Virginia Macdonald

**Affiliations:** 1 Department of International Health Johns Hopkins Bloomberg School of Public Health Baltimore, MD United States; 2 Testing, Prevention and Population Unit Global HIV, Hepatitis and STI Programmes World Health Organization Geneva Switzerland; 3 Regional Support Team Asia Pacific Joint United Nations Programme on HIV/AIDS New Delhi India

**Keywords:** online service delivery, digital health interventions, HIV, viral hepatitis, sexually transmitted infections, key populations, systematic review, mobile phone

## Abstract

**Background:**

Despite the growth of web-based interventions for HIV, viral hepatitis (VH), and sexually transmitted infections (STIs) for key populations, the evidence for the effectiveness of these interventions has not been reported.

**Objective:**

This study aimed to inform the World Health Organization guidelines for HIV, VH, and STI prevention, diagnosis, and treatment services for key populations by systematically reviewing the effectiveness, values and preferences, and costs of web-based outreach, web-based case management, and targeted web-based health information for key populations (men who have sex with men, sex workers, people who inject drugs, trans and gender-diverse people, and people in prisons and other closed settings).

**Methods:**

We searched CINAHL, PsycINFO, PubMed, and Embase in May 2021 for peer-reviewed studies; screened abstracts; and extracted data in duplicate. The effectiveness review included randomized controlled trials (RCTs) and observational studies. We assessed the risk of bias using the Cochrane Collaboration tool for RCTs and the Evidence Project and Risk of Bias in Non-randomized Studies of Interventions tools for non-RCTs. Values and preferences and cost data were summarized descriptively.

**Results:**

Of 2711 records identified, we included 13 (0.48%) articles in the effectiveness review (3/13, 23% for web-based outreach; 7/13, 54% for web-based case management; and 3/13, 23% for targeted web-based health information), 15 (0.55%) articles in the values and preferences review, and 1 (0.04%) article in the costs review. Nearly all studies were conducted among men who have sex with men in the United States. These articles provided evidence that web-based approaches are as effective as face-to-face services in terms of reaching new people, use of HIV, VH, and STI prevention services, and linkage to and retention in HIV care. A meta-analysis of 2 RCTs among men who have sex with men in China found increased HIV testing after web-based outreach (relative risk 1.39, 95% CI 1.21-1.60). Among men who have sex with men in the United States, such interventions were considered feasible and acceptable. One cost study among Canadian men who have sex with men found that syphilis testing campaign advertisements had the lowest cost-per-click ratio on *hookup* platforms compared with more traditional social media platforms.

**Conclusions:**

Web-based services for HIV, VH, and STIs may be a feasible and acceptable approach to expanding services to key populations with similar outcomes as standard of care, but more research is needed in low-resource settings, among key populations other than men who have sex with men, and for infections other than HIV (ie, VH and STIs).

## Introduction

### Background

Access to the internet and social media apps has grown exponentially in recent years, including in low-income settings and among vulnerable populations [1-4]. The potential advantages of providing health-related services on the web include reaching a broader audience; reaching people who are geographically isolated or who may not seek community or clinic-based services; targeting information to different groups and individuals; improving financial and system efficiencies; reducing stigma and discrimination; and offering clients greater anonymity, convenience, and potential self-care [5]. Thus, services are increasingly “going online,” including services designed for key populations who are disproportionately affected by HIV, viral hepatitis (VH), and sexually transmitted infections (STIs)—men who have sex with men, sex workers, people who inject drugs, trans and gender-diverse people, and people in prisons and other closed settings.

Web-based health interventions can take a wide variety of forms [6]. For key populations, common strategies include web-based outreach, web-based case management, and targeted web-based health information. Web-based outreach seeks to identify potential key population service users through web-based platforms, such as websites and social media apps, where key populations communicate, learn information, and socialize. Web-based case management can support key populations who have tested positive and need to engage in services to assess risk and adhere to necessary treatment as well as those who have tested negative and need counseling or biomedical prevention options such as preexposure prophylaxis or continued regular self-testing. Providing case management through web-based systems could potentially reduce loss to follow-up and provide behavioral nudges (such as reminders to book an appointment, take a test, or take a medication). Targeted web-based health information uses internet sites and social networking apps to target communication according to user demographics and characteristics. For example, Facebook advertisements can target users of certain ages, social profiles, geographic locations, or other attributes. Population segmentation may allow for more specific targeting of key population audiences to tailored information or linkage to health services.

### Objective

Despite the growth of web-based interventions for key populations, particularly accelerated during the COVID-19 pandemic, there has not been a synthesis of the effectiveness of these strategies across key populations. A review of digital health interventions addressing sexual risk, substance use, and common mental health conditions among men who have sex with men [7,8] found such interventions to be acceptable across sociodemographic groups and usually based on individual-level theoretical constructs such as self-efficacy, motivation, behavioral intentions, attitudes, and perceived norms. We identified no similar reviews on digital health interventions with sex workers, people who inject drugs, people in prisons and other closed settings, or trans and gender-diverse populations. To inform the World Health Organization (WHO) guidelines for HIV, VH, and STI service delivery for key populations, we systematically reviewed the effectiveness, values and preferences, and costs of web-based outreach, web-based case management, and targeted web-based health information.

## Methods

### Overview

This systematic review addressed the following question: *Does providing services on the web improve uptake of HIV, VH, and STI prevention, testing, linkage to treatment, and treatment retention for key populations?* We reviewed the extant literature in 3 related areas, which are components of the evidence-based process used to inform WHO guideline development [9]: (1) effectiveness of the intervention, (2) values and preferences of end users and health workers related to the intervention, and (3) cost information. We followed the PRISMA (Preferred Reporting Items for Systematic Reviews and Meta-Analyses) guidelines [10].

### Ethics Approval

Ethics approval was not required for this systematic review as all the data were obtained from published articles.

### Effectiveness Review: Eligibility Criteria

We designed the effectiveness review according to the PICO (population, intervention, comparison, and outcomes) format.

#### Population

The population of interest for the review includes (as described in the Glossary section of the 2022 WHO consolidated guidelines on HIV, VH, and STI prevention, diagnosis, treatment, and care for key populations [11]) men who have sex with men, sex workers, people who inject drugs, trans and gender-diverse people, and people in prisons and other closed settings.

#### Intervention

Given the range of web-based health interventions, the effectiveness review was split into 3 separate categories of web-based interventions: web-based outreach, web-based case management, and targeted web-based health information. We excluded noninternet phone-based (SMS text messaging or telephone call) outreach and case management approaches, counseling interventions, and general informational and educational videos about HIV, VH, and STI prevention. We excluded studies that used web-based methods to recruit study participants or to deliver the intervention of interest (eg, HIV self-testing) but did not compare service delivery modalities in their outcomes. We also excluded web-based service delivery interventions that did not specifically target key populations, even if many of their users were members of key populations, if the data were not disaggregated to specific key populations. The following definitions were used:

- *Outreach for HIV, VH, and STI services through web-based platforms* was defined as outreach conducted in any way through the internet (website or app, accessed through any device). Web-based outreach aims to identify and reach key populations who have previously not had contact with health services. Outreach can be active or passive, including reaching out to potential clients for information and linking them to services such as referrals, follow-up reminders, and counseling. These outreach approaches may include web-based peer-to-peer networks, social media influencers, and advertisements. These interventions aim to reach populations using social media platforms and dating apps, who do not necessarily identify themselves as key populations but are equally vulnerable and need to be linked to services across the cascade.
- *Web-based case management* was defined as web-based methods for case managers to conduct risk screening, referral, partner notification, appointment scheduling, and reminders for prevention, testing and treatment services, treatment adherence support, follow-up, counseling, telemedicine, and home delivery of services.
- *Targeted web-based health information* was defined as a web-based awareness generation or demand creation method. These targeted approaches deliver information and education, behavior change communication, or health service advertisements through mechanisms such as Facebook and dating apps, where data can be mined via algorithms to target specific messages to different key population groups (population segmentation and microtargeting). For example, targeted messages that aim to bring about behavioral change by motivating or mobilizing followers to get tested or learn about prevention can be passed through social media influencers.

#### Comparator

The comparator group (for each of the 3 intervention categories) was standard of care.

#### Outcomes

The outcomes of interest for each intervention category are presented in Table 1.

**Table 1:** Outcomes of interest by category of web-based service delivery.

	Web-based outreach	Web-based case management	Targeted web-based health information
Number or proportion of previously unreached people reached	✓		
Use of prevention services (eg, PrEP^a^ uptake, PrEP adherence, PEP^b^ uptake, PEP adherence, counseling, and condoms)	✓	✓	✓
Uptake of testing services for HIV, VH^c^, and STIs^d^	✓	✓	✓
Treatment initiation for HIV, VH, and STIs	✓	✓	✓
Treatment retention or completion for HIV, VH, and STIs		✓	✓
Viral load (eg, HIV and HCV^e^) testing or suppression		✓	✓
Cure (for curable STIs, eg, HCV, syphilis, and gonorrhea)		✓	✓
Mortality		✓	✓

^a^PrEP: preexposure prophylaxis.

^b^PEP: postexposure prophylaxis.

^c^VH: viral hepatitis.

^d^STI: sexually transmitted infection.

^e^HCV: hepatitis C virus.

To be included in the effectiveness review, an article must have (1) had a study design that compared web-based service delivery with standard of care for key populations, including randomized controlled trials (RCTs), non-RCTs, and comparative observational studies (including prospective controlled cohort studies, retrospective controlled cohort studies, cross-sectional studies, controlled before-after studies, and interrupted time series) that compared individuals who received the intervention with those who did not; (2) measured ≥1 of the outcomes of interest for that category of interventions; and (3) been published in a peer-reviewed journal between January 1, 2010, and the search date of May 27, 2021. No restrictions were placed based on location of the intervention or language of the publication.

### Search Strategy and Screening

We searched 4 databases (CINAHL, PsycINFO, PubMed, and Embase) for relevant peer-reviewed publications. Search terms covered terms for key populations, infections (HIV, VH, STIs), and web-based service delivery interventions. The full search strategy is presented in Multimedia Appendix 1. This search was complemented by several other methods of identifying articles. First, we ran 2 earlier searches for behavioral interventions for key populations more broadly—one in 2020 as part of a scoping review and one in March 2021 as part of a search only for RCTs. Articles identified through these prior searches were included in the review. Second, we hand searched the references of articles identified for inclusion in the review. Third, we contacted experts in the field (including members of the WHO Key Population Guideline Development Group) to identify any additional articles that we may have missed.

Titles, abstracts, citation information, and descriptor terms of citations identified through the search strategy were screened for initial inclusion. Full-text articles were obtained of all selected abstracts, and 2 independent reviewers assessed all full-text articles for eligibility to determine final study selection. Differences were resolved through consensus.

### Data Management and Analysis

Two reviewers independently abstracted data using standardized forms in Microsoft Excel (Microsoft Corporation). Differences in data abstraction were resolved through consensus and referral to a senior study team member as necessary. We collected the following information from each article: study identification (author, year, title, journal, and language of article), location (country, urban or rural, World Bank income classification, and WHO region), key population description (gender and age), sample size (n), study design (including follow-up periods and loss to follow-up), intervention description (including who delivered intervention, where intervention was provided, and how long or frequent intervention was), comparator description, and study outcomes (analytic approach, outcome measures or definitions, intervention vs comparison group, frequency and percentage or effect sizes with CIs or significance levels, conclusions, and limitations).

For RCTs, risk of bias was assessed using the Cochrane Collaboration’s tool for assessing risk of bias [12]. Methodological components of the studies were assessed and classified as high or low risk of bias. For studies that were not randomized trials but were comparative, study rigor was assessed using the Evidence Project 8-item checklist for intervention evaluations [13] and Risk of Bias in Non-randomized Studies of Interventions [14].

Data were analyzed according to the coding categories and outcomes. Where there were multiple studies reporting the same outcome for the same intervention-comparator comparison for the same population, we conducted meta-analysis using Comprehensive Meta-Analysis software (Biostat Inc). All outcomes were stratified and presented by key population. Findings were summarized in Grading of Recommendations, Assessment, Development, and Evaluations (GRADE) evidence profile tables using GRADEPro, prioritizing RCT data over observational data where available.

### Values and Preferences Review

The same search terms were used to search and screen for studies to be included in the values and preferences review. Studies were included in this review if they presented primary data examining the values and preferences of potential beneficiaries, communities, providers, and stakeholders for web-based service delivery interventions. These studies could be qualitative or quantitative in nature but had to present primary data collection; think pieces and review articles were not included. Values and preferences literature was summarized qualitatively and was organized by study design and methodology, location, and population.

### Cost and Resource Needs

The same search terms were used to search and screen for studies to be included in the cost review. Studies were included in this review if they presented primary data comparing cost, cost-effectiveness, cost utility, or cost-benefit of the intervention and comparison listed in the PICO question or if they presented cost-effectiveness of the intervention as it relates to the PICO outcomes listed in Table 1. We qualitatively summarized the cost literature. We organized the cost literature into 4 categories (health sector costs, other sector costs, patient or family costs, and productivity impacts) and presented it by study design or methodology, location, and population within each category.

## Results

### Overview

**Figure 1:**
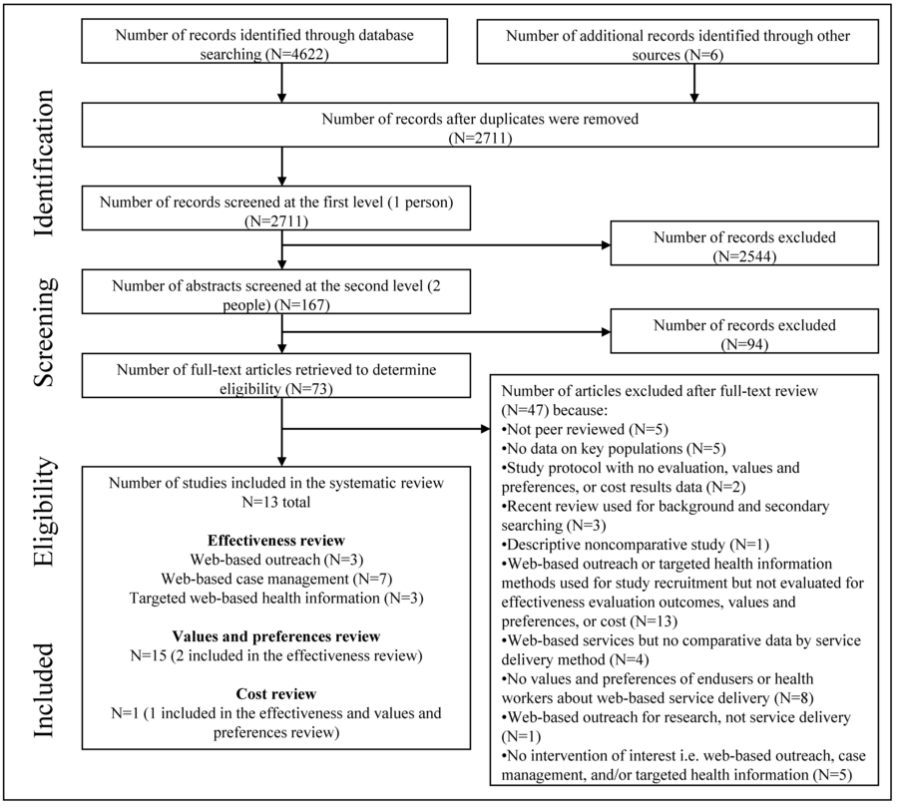
PRISMA (Preferred Reporting Items for Systematic Reviews and Meta-Analyses) flowchart depicting the search and screening process.

Our database search yielded 4622 records, and we identified another 6 through hand searching and secondary searching (Figure 1). Of the 2711 unique records, 73 (2.69%) were retained for full-text review. Ultimately, we included 3 articles in the effectiveness review on web-based outreach [15-17], 7 articles in the effectiveness review on web-based case management [18-24], 3 articles in the effectiveness review on targeted web-based health information [25-27], 15 articles in the values and preferences review [24,27-40], and 1 article in the costs review [27].

### Effectiveness Review

A total of 2 RCTs [16,17] and 1 serial cross-sectional study [15], all among men who have sex with men, were included in the effectiveness review of web-based outreach; 4 RCTs [20,21,23,24] and 3 observational studies [18,19,22] among men who have sex with men, trans and gender-diverse people, and people in prisons and other closed settings were included for web-based case management; and 2 RCTs [25,26] and 1 observational study [27], all among men who have sex with men, were included for targeted web-based health information. Descriptive data for the included articles are presented in Table 2, risk of bias assessments are presented in Multimedia Appendix 2 [15-27], and findings by PICO outcome within each intervention category in GRADE evidence profiles are presented in Multimedia Appendix 3 [15-27].

**Table 2:** Description of studies included in the effectiveness review.

Study; design		Population; sample size; location	Disease focus	Intervention	Comparison	Outcomes
**Web-based outreach**						
	Tang et al [16], 2018; stepped-wedge cluster RCT^a^	MSM^b^; N=1381; China	HIV and STIs^c^	Integrated web-based HIV testing: an HIV testing intervention was developed through a national image contest, a regional strategy design-a-thon, and local message contests. The final intervention included a multimedia HIV testing campaign, a web-based HIV testing service, and local testing promotion campaigns tailored for MSM.	Conventional HIV testing programs routinely provided by local centers for disease control and community-based organizations	HIV testing and syphilis testing
	Zhu et al [17], 2019; RCT	MSM; N=100; China	HIV and STIs	Web-based HIV self-testing via WeChat: intervention participants received 2 oral HIV self-testing kits and 6-month access to WeTest (a private WeChat group that provided app-based messages and referrals to HIV services, ie, brief informational messages on HIV, STIs, and HIV testing; first-person stories about people diagnosed and living with HIV; local data about HIV and STI among MSM; news about national policies related to HIV; stories about general health concerns of MSM; video or textual information about using the oral HIV self-testing kit; and 2-way communication between participant and WeTest team).	Brief video about self-administering the oral HIV self-testing kit (baseline procedures only)	HIV self-testing
	Lampkin et al [15], 2016; serial cross-sectional	MSM; N=NR^d^; United States	HIV and STIs	Outreach via Grindr: suburban public health department in San Mateo County, California, used a social networking smartphone app designed for gay and bisexual men as a platform to engage MSM in STI outreach, education, and testing (screening and linkage to care).	Pre-Grindr outreach implementation	Total contacts with MSM by public health department, HIV primary care, and HIV and STI testing
**Web-based case management**						
	Arayasirikul et al [18], 2020; prepost	MSM and TG^e^ (HIV+, aged 18-34 years); N=120; United States	HIV	Six-month digital HIV care navigation intervention (connected to personal HIV care navigator via SMS text messaging) to improve engagement in HIV care.	Did not complete intervention	Received HIV primary care, currently taking ART^f^, and viral suppression
	Brantley et al [19], 2019; cohort	PRIS^g^ (HIV+ adults soon to be released); N=238; United States	HIV	Web-based, tailored HIV and STI testing intervention, with baseline assessment and access to a tailored, personalized website.	Web-based provider directory page only	HIV and STI testing
	Horvath et al [23], 2020; RCT	MSM (HIV−); N=113; United States	HIV	Mobile app “SUP^h^” for repeat HIV testing (monthly My Health Survey to recommend next test date; prevention “411” directory with HIV and STI information).	No treatment	HIV testing
	Horvath et al [24], 2019; RCT	MSM (HIV+ stimulant users); N=90; United States	HIV	Mobile app “APP+” to improve ART adherence (IMB^i^ HIV and ART content, choose-your-own-adventure story, and medication self-monitoring).	No treatment	ART adherence
	Kuo et al [20], 2019; RCT	PRIS (HIV+ adults soon to be or recently released); N=110; United States	HIV	“CARE+Corrections” program to enhance HIV care engagement (computerized motivational interview or individual risk reduction plan) and SMS text messaging after release.	Opioid overdose prevention video and HIV providers or resources printout	Engagement in HIV care
	Songtaweesin et al [21], 2020; RCT	MSM and TG (HIV−, aged 15-19 years); N=200; Thailand	HIV	Mobile app with youth‐friendly services plus a mobile PrEP^j^ app (self-assessment of HIV risk, rewards, and reminders for PrEP and clinic appointments).	Mobile app with youth-friendly services only	PrEP adherence
	Young et al [22], 2014; cohort	PRIS (HIV+); N=1201; United States	HIV	Telemedicine (managed by a university-based multidisciplinary subspecialty team via a telemedicine clinic).	On-site management by correctional facility physicians	Viral suppression
**Targeted web-based health information**						
	Bauermeister et al [26], 2015; RCT	MSM; N=130; United States	HIV and STIs (hepatitis A virus, hepatitis B virus, human papilloma virus, chlamydia, gonorrhea, and syphilis)	The tailored website included content customized based on prior testing experiences and motivations, barriers and resources to testing, and important values (gathered during baseline assessment). These personalized messages were included in web-based content, for example: STI facts; personal motivations, values, and strengths assessment regarding STI testing; exploration of barriers (eg, ﬁnancial costs, social norms, and prioritization) to the participant’s desire to get tested for HIV and STIs; and a listing of providers.	Access to the web-based provider directory (no personalized or tailored content)	Hepatitis A virus, hepatitis B virus, and human papilloma virus vaccination and HIV and STI testing
	Young et al [25], 2013; RCT	MSM; N=112; United States	HIV	Project Harnessing Online Peer Education: social networking or peer leaders on Facebook to deliver information on HIV prevention or discuss HIV-related topics both individually and as a group via chat, wall posts, and personal messages over 12 weeks.	Peer leaders on Facebook to deliver information on general health	HIV testing
	Ross et al [27], 2016; serial cross-sectional	MSM; N=NR; Canada	STIs (syphilis)	The Winnipeg Regional Health Authority developed a campaign in 2014 highlighting the syphilis outbreak and the importance of seeking testing. Over 1 month, advertisements appeared on 4 web-platforms: Grindr, Facebook, Squirt, and the Gay Ad Network. When clicked, the advertisements would direct the user to an information website.	Precampaign	Syphilis testing

^a^RCT: randomized controlled trial.

^b^MSM: men who have sex with men.

^c^STI: sexually transmitted infection.

^d^NR: not reported.

^e^TG: trans and gender-diverse people.

^f^ART: antiretroviral therapy.

^g^PRIS: people in prisons and other closed settings.

^h^SUP: Status Update Project.

^i^IMB: Information-Motivation-Behavioral.

^j^PrEP: preexposure prophylaxis.

### Web-Based Outreach

One stepped-wedge RCT among 1381 men who have sex with men in China compared integrated web-based HIV testing (multimedia HIV testing campaign, web-based HIV testing service, and local testing promotion campaigns tailored for men who have sex with men) with conventional HIV testing routinely provided by local centers for disease control and community-based organizations [16]. Another RCT among 100 men who have sex with men in China compared web-based HIV self-testing via WeChat promotion or referrals with watching a brief video about self-administering the oral HIV self-testing kit [17]. A serial cross-sectional study among men who have sex with men in the United States compared using Grindr for STI outreach, education, and screening or linkage to care with standard of care [15].

Two studies reported the impact of web-based outreach on condom use. A stepped-wedge cluster RCT among men who have sex with men in China found little or no difference in self-reported condom use, comparing clusters who received the web-based HIV testing intervention with conventional HIV testing programs routinely provided by local centers of disease control and community-based organizations (relative risk [RR] 1.00, 95% CI 0.86-1.17); with little risk of bias, this RCT provided high-certainty evidence of no effect [16]. Another RCT of men who have sex with men in China provided low-certainty evidence that web-based outreach may make little or no difference to self-reported condom use, regardless of partner type (RR 0.90, 95% CI 0.39-2.06) [17].

The same 2 RCTs also reported on infection testing among men who have sex with men in China. A meta-analysis provided moderate-certainty evidence that the interventions increased HIV testing (RR 1.39, 95% CI 1.21-1.60) [16,17]. One RCT provided moderate-certainty evidence of probably no difference in syphilis testing (RR 0.92, 95% CI 0.70-1.21) [16].

One serial cross-sectional study among men who have sex with men in the United States provided moderate-certainty evidence that the intervention probably reached more previously unreached people [15]. When only traditional outreach methods were used (October 2011 to March 2012), the local public health department had contact with 60 men who have sex with men. After implementing outreach via Grindr (October 2013 to March 2014), the department had contact with 816 men who have sex with men. There was no denominator to calculate the rates and possible confounding from other factors, creating a potential risk of bias.

No studies provided comparative outcomes on other measures of the use of prevention services or on treatment initiation.

### Web-Based Case Management

A total of 3 RCTs [20,23,24] and 3 comparative observational studies [18,19,22] examining web-based case management were conducted in the United States, and an additional RCT was conducted in Thailand [21]. Furthermore, 4 studies were conducted among men who have sex with men [18,21,23,24] (2 also included transgender women [18,21]), and 3 studies were conducted among people in prisons and other closed settings [19,20,22] (2 of which examined postrelease linkage or engagement in care [19,20]). All interventions were designed for web-based case management of primarily HIV services, whether in terms of engagement in preexposure prophylaxis services [21], HIV and STI testing [19,23], or linkage to care, antiretroviral therapy adherence, and HIV care engagement [18,20,22,24].

One RCT among men who have sex with men and transgender women in Thailand provided low-certainty evidence that web-based case management probably had no effect on the use of prevention services. This study compared preexposure prophylaxis adherence among those who used a web-based case management app with those who did not (RR 1.12, 95% CI 0.78-1.59) [21].

One RCT among men who have sex with men in the United States showed no difference in the uptake of repeat HIV testing with web-based case management (RR 1.24, 95% CI 0.78-1.95) [23] but with very low certainty.

A cohort study among prisoners living with HIV in the United States found that there may be no difference in linkage to care after release from prison, comparing those who used a web-based tailored or personalized website with those who only had access to a web-based provider directory web page (RR 1.09, 95% CI 0.92-1.29; low-certainty evidence) [19]. Another cohort study among men who have sex with men and transgender women in the United States found a modest increase in the proportion of those who received primary HIV care in the last 6 months, comparing those who completed a 6-month digital HIV care navigation intervention with those who did not (RR 1.20, 95% CI 1.01-1.42; low-certainty evidence) [18].

One RCT in the United States among 90 men who have sex with men, who are living with HIV, and who use stimulants provided moderate-certainty evidence of probably higher overall antiretroviral therapy adherence in the intervention than in the control arm at 4 months (intervention: self-reported percentage ART adherence in the past 30 days 89% [95% CI 83.4-94.6] vs control: 77.2% [95% CI 66.7-87.7]; difference: 11.8% [95% CI 0.34-23.2]; *P*=.04), but 2 months later at 6 months, the improvements in adherence had probably dissipated (intervention: 85.3% [95% CI 80.0-90.6] vs control: 89.0% [95% CI 83.2-94.9]; difference: −3.7% [95% CI −11.4 to 4.0]; *P*=.34) [24].

One RCT among 110 people living with HIV who were recently incarcerated in the United States provided low-certainty evidence that web-based outreach may make little or no difference in engagement in HIV care, measured by having seen an HIV care provider in the community at least once in the past 24 weeks (RR 0.98, 95% CI 0.85-1.12) [20]. A cohort study among 120 men who have sex with men and transgender women living with HIV in the United States provided low-certainty evidence that web-based case management may make little to no difference in the proportion of people in each group self-reporting currently taking antiretroviral therapy (RR 1.19, 95% CI 0.97-1.45) [18].

The RCT among recently incarcerated persons living with HIV in the United States also provided low-certainty evidence that receiving a computerized motivational interview and individual risk reduction plan prerelease plus SMS text messaging about care navigation after release may make little or no difference to HIV viral suppression (laboratory-assessed viral load <200 copies/mL; RR 0.97, 95% CI 0.69-1.36) [20]. Similarly, a cohort study among men who have sex with men and transgender women living with HIV in the United States found that using web-based case management was associated with little or no difference in viral suppression (self-reported viral load <200 copies/mL; RR 1.05, 95% CI 0.79-1.40) [18], but another cohort study among 1201 incarcerated persons in the United States provided moderate-certainty evidence of probable improvement in the proportion of participants who had laboratory-assessed HIV virologic suppression at any of their first 6 care visits (RR 1.53, 95% CI 1.43-1.64) [22].

No studies measured our other outcomes of cure or mortality.

### Targeted Web-Based Health Information

None of the included articles precisely fit our topic definition for “targeted web-based health information,” but 3 articles were close enough to give a general idea of its effectiveness; the observational study may have been the closest to the intervention we desired to evaluate. This serial cross-sectional study among men who have sex with men in Winnipeg, Canada, compared syphilis testing rates before and after a social media campaign highlighting the syphilis outbreak and the importance of seeking testing, where advertisements on 4 web-based platforms (Grindr, Facebook, Squirt, and the Gay Ad Network) brought users to an information website connecting them with testing [27]. One RCT among 130 men who have sex with men in the United States compared participants who had access to a website customized to the user based on their personal prior testing experiences and motivations, barriers, and resources to testing with those who received access to a web-based provider directory with no tailored content [26]. Another RCT among 112 men who have sex with men in the United States compared having social networks or peer leaders on Facebook delivering HIV information in group settings and individually (via chat, wall posts, and personal messages) with peer leaders on Facebook delivering general health information [25].

One RCT among men who have sex with men in the United States provided low-certainty evidence that targeted web-based health information may make little or no difference in HIV testing (following up for HIV test result after requesting an HIV self-test kit and returning the kit; RR 3.56, 95% CI 0.32-39.65), although more participants in the intervention arm requested the kit than those in the control arm [25]. A serial cross-sectional study among men who have sex with men in Canada provided low-certainty evidence of little or no difference in the number of people who ordered a syphilis test before, during, or after a syphilis testing web-based advertisement campaign (RR 1.00, 95% CI 0.94-1.07) [27]. Another RCT among men who have sex with men in the United States found no impact of targeted web-based health information on HIV and STI testing (RR 1.46, 95% CI 0.72-2.94), although the sample size was small and the certainty of evidence was very low [26].

One RCT among men who have sex with men in the United States provided very low-certainty evidence on whether targeted web-based health information improves uptake of prevention services: no vaccinations for STIs were conducted in either arm, but sample sizes were very small (68 in the intervention arm and 36 in the control arm) [26].

No studies measured other outcomes of interest: treatment for HIV, VH, and STIs, treatment retention or completion for HIV, VH, and STIs, viral load testing or suppression, cure, or mortality.

### Values and Preferences Review

Three cross-sectional studies examined the values and preferences around web-based outreach for key populations. These were conducted among men who have sex with men in Spain [36], men who have sex with men in China [40], and people who self-identified as a sexual or gender minority in East Africa [39]. Another 7 studies—all among men who have sex with men in the United States—examined values and preferences around web-based case management, sometimes in the context of assessing pilot programs [24,30-35]. Three studies in Canada, England, and the United States explored values and preferences around targeted web-based health information, all among men who have sex with men [27-29]. Specific data from each included article in the values and preferences review are presented in Multimedia Appendix 4 [24,27-40].

Regarding web-based outreach, in Spain, men who have sex with men thought it was acceptable to receive unsolicited messages about rapid HIV, syphilis, and hepatitis C testing on social media or *hookup* apps [36]. In Uganda, Tanzania, Rwanda, South Sudan, and Kenya, almost half of the survey respondents who self-identified as a sexual or gender minority were *very likely* to engage in a sexual health program if outreach was conducted on the web, over SMS text message, or over email [39]. In China, men who have sex with men reported high interest and willingness to use a “men who have sex with men-friendly physician finder function” within gay mobile apps [40].

Regarding web-based case management, men who have sex with men in China expressed interest in features and functions related to sexual health that could be embedded into existing smartphone apps or developed as stand-alone apps [35]. Men who have sex with men in the United States were strongly in favor of a smartphone app developed for web-based case management [31]. Positive features of such apps included their ease of use (eg, easy to navigate, fast, and convenient), the ability to set reminders or alarms to take medication at a certain time each day, trackers for adherence, and communication with providers, which helped users feel supported in their care process. However, several studies mentioned concerns about ensuring confidentiality in the web-based environment [24,30,31].

When asked about targeted web-based health information specifically, men who have sex with men expressed a diverse range of acceptability, ranging from indifference to frustration to enabling care seeking to influencing risk behaviors, and most were comfortable interacting with health services on the web, including through platforms that are not typically for health services, such as geosocial networking sites, for example, Facebook, or dating apps, for example, Grindr [27-29]. One study found that targeted web-based health information provided through *hookup sites* may garner more interest or be more acceptable than standard social media [27].

In 2 qualitative studies among frontline outreach workers, managers, or public health volunteers who worked with men who have sex with men in Canada [37,38], participants said that web-based technologies have reshaped the *gay or queer community*, have changed norms for social or sexual interactions, and can help reach out to hard-to-reach people. These studies found that web-based outreach generally allowed for more nonintrusive and anonymous communication (beneficial for clients), and that quick feedback helped them to be responsive to user needs. Respondents also noted some barriers to web-based outreach, such as quality of service, collaborations between outreach service agencies and companies that own apps and websites, budgetary and staff or volunteer capacity constraints, and data security and safety. On the basis of their experience, service providers described 4 ethical dilemmas as outreach moved to web-based platforms: (1) managing personal or professional boundaries with clients, (2) disclosing personal or identifiable information to clients, (3) maintaining client confidentiality and anonymity, and (4) security and data storage measures of web-based information.

### Cost Review

Only 1 study was identified for the cost review [27]. This study among men who have sex with men in Canada found that syphilis testing campaign advertisements had the lowest cost-per-click ratio on the *hookup* platforms Grindr and Squirt, compared with more traditional social media platforms such as Facebook and the Gay Ad Network. No studies measured cost-effectiveness.

## Discussion

### Principal Findings

Overall, our effectiveness review found comparable outcomes when using web-based service delivery methods compared with standard of care, indicating that web-based approaches may be at least as effective as face-to-face services in terms of reaching new people, use of HIV and STI prevention services, and linkage to and retention in HIV care. However, the certainty of evidence for many outcomes in the effectiveness review was generally moderate or low, and for several PICO outcomes, either no statistically significant effect was reported or no studies measured the outcome of interest. These findings are broadly similar to those of other systematic reviews of digital health interventions with diverse health topics and populations [41-43]. When considering the values and preferences of end users and health workers, we generally found that web-based services for key populations were feasible and acceptable, similar to findings from other more general systematic reviews [44-46]. One cost study found that targeted messaging using web-based dating platforms may reach more end users than traditional web-based social media platforms. Programmatic descriptions from FHI 360’s “Going online budgeting guide” designed to help accelerate the impact of HIV programs [47] show a wide range of costs for programs, depending on scope of work, including but not limited to country or regional costs, connectivity level, program intensity or scale, vendors, in-person trips or training needs, and equipment needs.

For some people who are unable to attend face-to-face services, web-based services may offer their only means of accessing information, support, referral, and case management. A study among youth found that web-based sexual health information is most valuable to youth who lack alternatives, and youth were more likely to take follow-up action if they had sought information for reasons related to privacy or having *no one to ask*, especially among gender minority youth [48]. However, some people have limited access to the internet, low reading ability, or low digital literacy; therefore, web-based services may not meet the needs of the most vulnerable. Potential harms relating to data security and confidentiality are of particular concern for key populations who may engage in criminalized and stigmatized activities and experience discrimination, arrest, or harassment if confidentiality is breached. These could also be issues for people who share devices, younger key populations, or groups such as sex workers who may change devices frequently. The ethics surrounding data mining are of special concern when targeting health information using social media platforms. However, capacity building of outreach staff and health workers regarding data security and confidentiality could mitigate this challenge. Other limitations to web-based services may include loss of in-person rapport and relationships when counseling and financial costs for end users (such as internet or airtime or for-profit apps).

Web-based services should be an additional, complementary approach within larger HIV, STI, and VH programs for key populations. Web-based services could be an important additional way to reach more people, reduce costs, reduce waiting times (by reducing clinic attendance), and allow more time for more complex case management in health facilities. Systematic planning on how web-based services can complement other services and target audiences should be conducted, with key populations playing a central role in design, implementation, and monitoring. Content development should be informed by appropriate and accurate health content and information aligned with recommendation practices (eg, from health program guidelines or evidence-based normative practices) and country policies for platform and language use [49]. In addition, maintenance of safety and security measures of web-based services will be required [50].

This review had several strengths. We conducted a comprehensive search across multiple databases as well as a hand search and secondary search. We assessed the methodological quality of the included articles and examined not only the effectiveness of web-based services for key populations but also end users’ and health workers’ values and preferences and costing. However, the available published research on web-based services for key populations was conducted mainly among men who have sex with men in high-income countries, and web-based service delivery options were usually limited to HIV prevention and treatment services. Among the included studies, we were only able to meta-analyze 1 outcome that provided comparable measurement methods for similar populations. Further research is needed on web-based services for other key populations in low- and middle-income settings and their effect on outcomes related to STI and VH. More research is needed on the cost-effectiveness of different types of web-based services for key population groups. Although many government- and community-based programs are already using web-based services for key populations, suggesting that it is a feasible intervention in many settings [51], these could not be included in our effectiveness review because no explicit comparison has been made between their web-based service delivery and in-person modalities. Finally, we excluded SMS text messaging interventions and other digital health interventions that were not primarily conducted on the web. Although we did this purposefully to narrow this review’s focus, we are not able to comment on the effectiveness, values and preferences, or costs related to these other approaches.

Using the evidence from this review and discussion among the experts in the WHO Key Population Guideline Development Group, the new consolidated WHO guideline for key populations includes a new conditional recommendation based on low-certainty evidence: “Online delivery of HIV, viral hepatitis, and STI services to key populations may be offered as an additional option while ensuring that data security and confidentiality are protected” [11].

Following this review’s search cutoff date and the WHO guideline development group meeting that led to the above recommendation, several study protocols [52,53] have been published for forthcoming RCTs, suggesting a growing evidence base for this topic.

### Conclusions

Web-based services for HIV, VH, and STIs are becoming increasingly common, especially in high-income settings, and are generally accepted by end users and health workers, suggesting their feasibility as an additional option for service delivery for key populations. Although largely acceptable, there will be people who do not wish to access web-based services and prefer face-to-face communication with health workers. This review of the extant peer-reviewed literature suggests that web-based service delivery may be a feasible and acceptable approach to expanding services to key populations with similar outcomes as standard of care; however, more research is needed on how to improve the effectiveness of these web-based service delivery interventions. Further research is also needed in low-resource settings, among key populations who are not men who have sex with men, and with infections other than HIV (ie, VH and STIs).

## Appendix

Multimedia Appendix 1 Search strategy for the systematic review of web-based service delivery for key populations.

Multimedia Appendix 2 Risk of bias assessments for articles included in the effectiveness review.

Multimedia Appendix 3 Grading of Recommendations, Assessment, Development, and Evaluations tables presenting the summary of evidence used for the effectiveness review.

Multimedia Appendix 4 Description of and key findings from the studies included in the values and preferences review.

## Abbreviations

GRADE: Grading of Recommendations, Assessment, Development, and Evaluations
PICO: population, intervention, comparison, and outcomes
PRISMA: Preferred Reporting Items for Systematic Reviews and Meta-Analyses
RCT: randomized controlled trial
RR: relative risk
STI: sexually transmitted infection
VH: viral hepatitis
WHO: World Health Organization
