# Supplementary material for: Web-Based Service Provision of HIV, Viral Hepatitis, and Sexually Transmitted Infection Prevention, Testing, Linkage, and Treatment for Key Populations: Systematic Review and Meta-analysis
Source: J Med Internet Res. 2022 Dec 22;24(12):e40150. doi: 10.2196/40150 (PMC9816952; doi:10.2196/40150)
Supplement: Multimedia Appendix 1 [file jmir_v24i12e40150_app1.pdf]

Appendix A. Search strategy for systematic review of online service delivery for key populations.

Pubmed

Concept 1: Key populations

Concept 1a: Sex workers
(Sex Workers [MeSH] OR Sex work [MeSH] OR sex work [tw] OR prostitut* [tw] OR commercial sex [tw] OR transactional sex [tw] OR SW [tw] OR FSW [tw] OR CSW [tw] OR sex trade [tw] OR trade sex [tw] OR sex industry [tw] OR entertainment worker [tw])

OR

Concept 1b: Men who have sex with men
(Homosexuality, Male [Mesh] OR gay men [tw] OR gay man [tw] OR gay male [tw] OR homosexual [tw] OR MSM [tw] OR men who have sex with men [tw] OR males who have sex with males [tw] OR bisexual men [tw] OR bisexual man [tw] OR bisexual male [tw] OR gay and bisexual men [tw] OR gay and other men who have sex with men [tw])

OR

Concept 1c: People who inject drugs
(Drug Users [Mesh] OR Substance Abuse, Intravenous [Mesh] OR drug use [tw] OR drug user [tw] OR drug users [tw] OR intravenous drug user [tw] OR injecting drug user [tw] OR injection drug user [tw] OR drug abuse* [tw] OR substance use [tw] OR substance abuse* [tw] OR people who inject drugs [tw] OR people who use drugs [tw] OR IVDU [tw] OR IDU [tw] OR PWUD [tw] OR PWID [tw] OR drug usage [tw])

OR

Concept 1d: Trans and gender diverse people
(Transgender Persons [Mesh] OR transgender [tw] OR transsexual [tw] OR transvest* [tw] OR travesti* [tw] OR trans [tw] OR koti [tw] OR hijra [tw] OR mahu [tw] OR waria [tw] OR katoey [tw] OR berdache [tw] OR muxe [tw] OR third spirit [tw] OR third spirited [tw] OR assigned male at birth [tw] OR assigned female at birth [tw] OR AMAB [tw] OR AFAB [tw] OR takataapui [tw] OR tahine [tw] OR whakawahine [tw] OR tangata ira wahine [tw] OR tangata ira tane [tw] OR irawhiti [tw] OR irahuhua [tw] OR MTF [tw] OR FTM [tw] OR transmasculine [tw] OR trans masculine [tw] OR transfeminine [tw] OR trans feminine [tw] OR people of trans experience [tw] OR non-binary [tw] OR nonbinary [tw] OR gender non-conforming [tw] OR genderqueer [tw] OR gender diverse [tw])

OR

Concept 1e: People living in prisons
(Prison [Mesh] OR Prisoners [Mesh] OR Criminals [Mesh] OR Concentration camps [Mesh] OR incarcerat* [tw] OR prison* [tw] OR jail* [tw] OR penitentiary [tw] OR penitentiaries [tw] OR

penal institution [tw] OR correctional center [tw] OR correctional centre [tw] OR correctional facility [tw] OR correctional facilities [tw] OR correctional setting [tw] OR detain* [tw] OR detention center [tw] OR detention centre [tw] OR inmate [tw] OR imprison* [tw])

OR

Concept 1f: general terms for key populations
(key population [tw] OR most at risk population [tw] OR MARPS [tw] OR vulnerable population [tw])

AND
Concept 2: HIV, VH, STIs

(HIV [Mesh] OR Acquired Immunodeficiency Syndrome [Mesh] OR HIV Infections [Mesh] OR human immunodeficiency virus [tiab] OR acquired immunodeficiency syndrome [tiab] OR HIV [tiab] OR AIDS [tiab] OR HIV1 [tiab] OR HIV2 [tiab] or Hepatitis, Chronic [Mesh] OR Hepatitis, Viral, Human [Mesh] OR hepatitis b [tiab] OR HBV [tiab] OR hepatitis c [tiab] OR HCV [tiab] OR hepatitis d [tiab] OR HDV [tiab] Or Sexually transmitted diseases [Mesh] OR STI [tiab] OR STD [tiab] OR sexually transmitted infection [tiab] OR sexually transmitted disease [tiab] OR sexually transmitted disorder [tiab] OR sexually transmissible infection [tiab] OR sexually transmissible disease [tiab] OR sexually transmissible disorder [tiab] OR anogenital wart [tiab] OR bacterial vaginosis [tiab] OR candida albicans [tiab] OR candidal vaginitis [tiab] OR candidiasis[tiab] OR candidosis [tiab] OR chancroid [tiab] OR chlamydia [tiab] OR Condylomata Acuminata [tiab] OR donovanosis [tiab] OR genital disorder [tiab] OR Gardnerella [tiab] OR genital infection [tiab] OR genital ulcer [tiab] OR genital wart [tiab] OR gonorrhea [tiab] OR gonorrhoea [tiab] OR Neisseria gonorrhoeae [tiab] OR granuloma inguinale [tiab] OR herpes [tiab] OR HPV [tiab] OR human papillomavirus [tiab] OR monilia albicans [tiab] OR monilial infection [tiab] OR syphilis [tiab] OR Treponema pallidum [tiab] OR trichomonas vaginalis [tiab] OR trichomoniases [tiab] OR trichomoniasis [tiab] OR venereal disease [tiab] OR venereal disorder [tiab] OR vulvitis [tiab] OR vulvovaginitis [tiab])

AND
Concept 3: Online outreach, online case management, or targeted online health information

(social influencer [tiab] OR social media [tiab] OR mhealth [tiab] OR ehealth [tiab] OR mobile health [tiab] OR digital health [tiab] OR technology-based [tiab] OR online [tiab] OR facebook [tiab] OR Instagram [tiab] OR twitter [tiab] OR grindr [tiab] OR dating site [tiab] OR dating app [tiab] OR social marketing [tiab] OR smartphone [tiab] OR internet [tiab] OR Web 2.0 [tiab] OR gamification [tiab] OR virtual reality [tiab] OR virtual [tiab] OR chat [tiab] OR chatbot [tiab] OR app [tiab] OR apps [tiab] NOT “online survey” [tiab] NOT “internet survey” [tiab]) OR ((mhealth [tiab] OR ehealth [tiab] OR mobile health [tiab] OR digital health [tiab] OR technology-based [tiab] OR smartphone [tiab] OR internet [tiab] OR Web 2.0 [tiab] OR telemedicine [tiab] OR EMR [tiab] OR electronic medical record [tiab] OR virtual [tiab] OR chat [tiab] OR chatbot [tiab] OR app [tiab] OR apps [tiab] OR online [tiab] AND (referral coordination [tiab] OR managed referrals [tiab] OR case management [tiab] OR continuity of patient care [MeSH] OR patient navigation [MeSH] OR patient-centered care [MeSH] OR case management [MeSH] OR case manager [MeSH] OR care coordinator [tiab] OR care coordination [tiab] OR care facilitator [tiab] OR care

facilitation [tiab] OR care navigator [tiab] OR care navigation [tiab] OR care advocate [tiab] OR care advocacy [tiab] OR care transition [tiab] OR care transitions [tiab] OR care peer [tiab] OR care peers [tiab] OR peer coach [tiab] OR service coordinator [tiab] OR service coordination [tiab] OR service navigator [tiab] OR service navigation [tiab] OR client facilitator [tiab] OR client navigation [tiab] OR client advocate [tiab] OR client advocacy [tiab] OR health coach [tiab] OR patient navigation [tiab] OR patient navigator [tiab] OR patient navigators [tiab] OR guided care [tiab] OR navigator [tiab] OR navigators [tiab] OR post-discharge support [tiab] OR peer navigator [tiab] OR peer navigators [tiab]))

CINAHL: Abstract only

Concept 1: Key populations

(Sex Workers OR Sex work OR sex work OR prostitut* OR commercial sex OR transactional sex OR SW OR FSW OR CSW OR sex trade OR trade sex OR sex industry OR entertainment worker) OR (Male homosexuality OR gay men OR gay man OR gay male OR homosexual OR MSM OR men who have sex with men OR males who have sex with males OR bisexual men OR bisexual man OR bisexual male OR gay and bisexual men OR gay and other men who have sex with men) OR (Drug Users OR Intravenous Substance Abuse OR drug use OR drug user OR drug users OR intravenous drug user OR injecting drug user OR injection drug user OR drug abuse OR substance use OR substance abuse OR people who inject drugs OR people who use drugs OR IVDU OR IDU OR PWUD OR PWID OR drug usage) OR (Transgender Persons OR transgender OR transsexual OR transvest* OR travesti* OR trans OR koti OR hijra OR mahu OR waria OR katoey OR berdache OR muxe OR third spirit OR third spirited OR assigned male at birth OR assigned female at birth OR AMAB OR AFAB OR takataapui OR tahine OR whakawahine OR tangata ira wahine OR tangata ira tane OR irawhiti OR irahuhua OR MTF OR FTM OR transmasculine OR trans masculine OR transfeminine OR trans feminine OR people of trans experience OR non-binary OR nonbinary OR gender non-conforming OR genderqueer OR gender diverse) OR (Prison OR Prisoners OR Criminals OR Concentration camps OR incarcerat* OR prison* OR jail* OR penitentiary OR penitentiaries OR penal institution OR correctional center OR correctional centre OR correctional facility OR correctional facilities OR correctional setting OR detain* OR detention center OR detention centre OR inmate OR imprison*) OR (key population OR most at risk population OR MARPS OR vulnerable population)

AND
Concept 2: HIV, Viral Hepatitis, STIs

(HIV OR Acquired Immunodeficiency Syndrome OR HIV Infections OR human immunodeficiency virus OR acquired immunodeficiency syndrome OR HIV OR AIDS OR HIV1 OR HIV2 OR Chronic Hepatitis OR Viral Hepatitis OR hepatitis b OR HBV OR hepatitis c OR HCV OR hepatitis d OR HDV Or Sexually transmitted diseases OR STI OR STD OR sexually transmitted infection OR sexually transmitted disease OR sexually transmitted disorder OR sexually transmissible infection OR sexually transmissible disease OR sexually transmissible disorder OR anogenital wart OR bacterial vaginosis OR candida albicans OR candidal vaginitis OR candidiasis OR candidosis OR chancroid OR chlamydia OR Condylomata Acuminata OR donovanosis OR genital disorder OR Gardnerella OR genital infection OR genital ulcer OR genital wart OR gonorrhea OR gonorrhoea OR Neisseria gonorrhoeae OR granuloma inguinale OR herpes OR HPV OR human papillomavirus OR monilia albicans OR monilial infection OR syphilis OR Treponema pallidum OR trichomonas

vaginalis OR trichomoniases OR trichomoniasis OR venereal disease OR venereal disorder OR vulvitis OR vulvovaginitis)

AND
Concept 3: Online outreach, online case management, or targeted online health information

(social influencer OR social media OR mhealth OR ehealth OR mobile health OR digital health OR technology-based OR online OR facebook OR Instagram OR twitter OR grindr OR dating site OR dating app OR social marketing OR smartphone OR internet OR Web 2.0 OR gamification OR virtual reality OR virtual OR chat OR chatbot OR app OR apps NOT “online survey” NOT “internet survey”) OR ((mhealth OR ehealth OR mobile health OR digital health OR technology-based OR smartphone OR internet OR Web 2.0 OR telemedicine OR EMR OR electronic medical record OR virtual OR chat OR chatbot OR app OR apps OR online) AND (referral coordination OR managed referrals OR case management OR continuity of patient care OR patient navigation OR patient-centered care OR case management OR case manager OR care coordinator OR care coordination OR care facilitator OR care facilitation OR care navigator OR care navigation OR care advocate OR care advocacy OR care transition OR care transitions OR care peer OR care peers OR peer coach OR service coordinator OR service coordination OR service navigator OR service navigation OR client facilitator OR client navigation OR client advocate OR client advocacy OR health coach OR patient navigation OR patient navigator OR patient navigators OR guided care OR navigator OR navigators OR post-discharge support OR peer navigator OR peer navigators))

PsycINFO: Abstract only

Search strategy same as CINAHL above

Embase (416 hits) TITLE/ABSTRACT/KEYWORDS ONLY

Search strategy same as CINAHL above
