# Supplementary material for: Web-Based Service Provision of HIV, Viral Hepatitis, and Sexually Transmitted Infection Prevention, Testing, Linkage, and Treatment for Key Populations: Systematic Review and Meta-analysis
Source: J Med Internet Res. 2022 Dec 22;24(12):e40150. doi: 10.2196/40150 (PMC9816952; doi:10.2196/40150)
Supplement: Multimedia Appendix 2 [file jmir_v24i12e40150_app2.pdf]

Appendix B. Risk of bias assessments for articles included in the effectiveness review.

Online outreach

**Cochrane Risk of Bias Tool (for RCTs)**							
**Risk of bias**	Risk of bias arising from the randomization process	Risk of bias due to deviations from the intended interventions		Risk of bias due to missing outcome data	Risk of bias in measurement of the outcome	Risk of bias in selection of the reported result	Overall risk of bias judgment
**Author Year**		Effect of assignment to intervention	Effect of adhering to intervention				
**Tang et al., 2018**	Low [1]	Low	Low	Low [2]	Low	Low	Low
**Zhu et al., 2019**	Low [3]	Low	Some concerns [4]	Low	Some concerns [5]	Low	Some concerns

**ROBINS-I Tool (for non-RCTs)**								
**Author Year**	Bias due to confounding	Bias in selection of participants into the study	Bias in classification of interventions	Bias due to deviations from intended intervention	Bias due to missing data	Bias in measurement of outcomes	Bias in selection of the reported result	Overall risk of bias judgment
**Lampkin et al., 2016**	Low [6]	Low	Low	Low	Moderate [7]	Low [8]	Low	Moderate

1 Although authors do not report specific details of the randomisation and/or allocation process are not documented, the study protocol states that 8 cities were randomized using SAS software. Because cities were randomized (not individuals), we did not judge there to be issues with blinding.

2 Loss to follow up between baseline and final follow-up assessment was > 90% but similar rates across arms.

3 Authors used a stratified block randomization scheme.

4 All participants in the intervention arm had the app downloaded onto their phones; levels of engagement with the app varied (i.e. viewing/opening WeTest messages).

5 Given the intervention of interest (online outreach), blinding was not possible for participants and personnel. However, deviations from the intended intervention due to lack of blinding were not documented, and any potential deviations were unlikely to have affected the outcome. However, the outcomes

(use of prevention services, use of testing services for HIV/VH/STIs) were judged as potentially influenced by lack of blinding since consistent condom use and HIV testing outcomes were self-reported.

6 While baseline confounding was not adjusted or, demographics and time-varying confounding were somewhat adjusted for (Grindr outreach between October 2012 and March 2013 was compared with the number of contacts initiated through traditional outreach activities during the same 6-month period from October 2011–March 2012; race and age were adjusted for in analysis). However, two years separated the two data collection time periods, and through use of Grindr for outreach was the main intervention, the public health dept may have adjusted other methods of reaching out to potential HIV/STI service users.

7 The outcome data not available for nearly all participants. Because the intervention (outreach via Grindr) was not obvious as research, retention rate after the outreach was identified as a health worker instead of a potential sexual partner was less than 80%, but engagement a secondary outcome after the primary outcome of outreach (# contacts made). There was no denominator (population) to calculate rates; only number of contacts - which may have included duplicates. For the uptake of prevention/testing services outcome, no comparisons to pre-Grindr outreach were reported.

8 Given the intervention of interest, blinding was not possible for personnel, whether blinding for participants was not mentioned in this study. Personnel are well-trained. The outcome measure was unlikely to be influenced by knowledge of the intervention received, the methods of outcome assessment comparable across intervention groups.

Online case management

**Cochrane Risk of Bias Tool (for RCTs)**							
**Risk of bias**		Risk of bias due to deviations from the intended interventions					
**Author Year**	Risk of bias arising from the randomization process	Effect of assignment to intervention	Effect of adhering to intervention	Risk of bias due to missing outcome data	Risk of bias in measurement of the outcome	Risk of bias in selection of the reported result	Overall risk of bias judgment
**Horvath et al., 2019a**	Some concerns [1]	Low [2]	Low [2]	Some concerns [3]	Some concerns [4]	Low	High
**Horvath et al., 2019b**	Low [5]	Low [2]	Low [2]	Low	Some concerns [4]	Low	Some concerns
**Kuo et al., 2019**	Some concerns [1]	Low [2]	Low [2]	Low	Low [4]	Low	Some concerns

**Songtaweesin et al., 2020**	Some concerns [1]	Low [2]	Low [2]	Low	Low [4]	Low	Some concerns	
**ROBINS-I Tool (for non-RCTs)**								
**Author Year**	Bias due to confounding	Bias in selection of participants into the study	Bias in classification of interventions	Bias due to deviations from intended intervention	Bias due to missing data	Bias in measurement of outcomes	Bias in selection of the reported result	Overall risk of bias judgment
**Arayasirikul et al., 2020**	Moderate [6]	Low	Low [7]	Low	Low	Moderate [4]	Low	Moderate
**Brantley et al., 2019**	Moderate [6]	Moderate [8]	Low	Low	Low	Low [4]	Low	Moderate
**Young et al., 2014**	Moderate [6]	Low	Low	Low	Low	Low [4]	Low	Moderate

1 Details of the randomisation and/or allocation process are not documented. No additional information on allocation concealment; some baseline differences between groups in terms of sociodemographics and outcome measures at baseline suggest potential issues.

2 Given the intervention of interest (online case mgmt), blinding was not possible for participants and personnel. However, no important non-protocol interventions were reported, and analyses were appropriate to estimate the effect of the intervention assignment.

3 By 8-month follow-up, loss-to-follow-up rate was 21% in the intervention arm and 12% in the control arm; differences in missingness could but unlikely depended on true value.

4 Given the intervention of interest (online case mgmt.), blinding was not possible for participants and personnel. However, deviations from the intended intervention due to lack of blinding were not documented, and any potential deviations were unlikely to have affected the outcome. We judged that self-reported outcomes (with no validation from lab/other measures) were potentially influenced by lack of blinding.

5 Authors “used a spreadsheet developed by the study statistician prior to the study launch to block randomize participants in a 1:1 allocation to the intervention or control arm”.

6 Baseline and time-varying confounding was not adjusted for measured validity and reliability by the variables available in the study.

7 Among the 120 participants enrolled in the intervention, 60 were lost to follow up and did not complete the intervention. The 60 who completed the intervention were considered the intervention group, and the 60 who were lost to follow up (67% for unknown reasons, 10% for phone loss, 15% for moving out of jurisdiction, and other reasons) were considered the control group. However, characteristics of the control group were not different from the overall sample (or the intervention group).

8 Selection of participants into the study was not based on participant characteristics observed after the start of the intervention, but the start of follow-up and start of intervention did not coincide for most participants (intervention group was those who successfully received the case management video conference in time for their release from prison; control group was those who did not receive the video conference in time).

Targeted online health information

**Cochrane Risk of Bias Tool (for RCTs)**							
**Risk of bias**	Risk of bias arising from the randomization process	Risk of bias due to deviations from the intended interventions		Risk of bias due to missing outcome data	Risk of bias in measurement of the outcome	Risk of bias in selection of the reported result	Overall risk of bias judgment
**Author Year**		Effect of assignment to intervention	Effect of adhering to intervention				
**Bauermeister et al., 2015**	Some concerns [1]	Some concerns [2]	Low [3]	Some concerns [4]	Low [5]	Low	Some concerns
**Young et al., 2013**	Low	Some concerns [6]	Low [3]	Low [7]	Low [5]	Low	Low

**ROBINS-I Tool (for non-RCTs)**								
**Author Year**	Bias due to confounding	Bias in selection of participants into the study	Bias in classification of interventions	Bias due to deviations from intended intervention	Bias due to missing data	Bias in measurement of outcomes	Bias in selection of the reported result	Overall risk of bias judgment
**Ross et al., 2016**	Moderate [8]	No information	Low	Low	Low	Moderate [9]	Serious [10]	Moderate risk of bias

1 Details of the randomisation and/or allocation process are not documented. The study says: “Next, participants were randomized into either the tailored experimental condition or the non-tailored control condition.” with no additional information on concealment or any baseline differences between groups.

2 Given the intervention of interest (targeted health information), blinding was not possible for participants and personnel. However, deviations from the intended intervention due to lack of blinding were not documented, and any potential deviations were unlikely to have affected the outcome. However, the outcomes (uptake of prevention services, uptake of testing services for HIV/VH/STIs) were judged as potentially influenced by lack of blinding. For uptake of prevention services, the outcome was measured by self-reported vaccination for hepatitis A or B, HPV, or meningococcal meningitis. For uptake of testing services for HIV/VH/STIs, the outcome was measured through self-reported testing by disease.

3 Given the intervention of interest (targeted health information), blinding was not possible for participants and personnel. However, no important non-protocol interventions were reported.

4 Outcome data at the 30-day follow-up assessment were reported for 80% of the 130 study participants (n=104). There was no evidence that the result was not biased by missing outcome data (e.g. analysis methods correcting for bias, sensitivity analyses), and missingness could depend on its true value, but we judged that missingness in the outcome was unlikely to depend on its true value because the loss to follow-up rate was similar across arms (68/86, 79% in the intervention arm and 36/44, 82% in the control arm).

5 The method of measuring the outcome was not inappropriate (HIV/STI testing and vaccination), and methods of measuring the outcome were comparable between groups.

6 Given the intervention of interest (targeted health information), blinding was not possible for participants and personnel. However, deviations from the intended intervention due to lack of blinding were not documented, and any potential deviations were unlikely to have affected the outcome. The outcome (uptake of testing services for HIV/VH/STIs) was judged as potentially influenced by lack of blinding. For uptake, the outcome was measured by self-reported uptake of HIV testing and subsequently verified by study/lab records (# kits requested, # kits returned, # followup for test kit result).

7 Though not an issue of missing outcome data among enrolled participants (n=112 across both arms, n=57 in the intervention arm and n=55 in the control arm – and a total of 105 participants (93.8%) completed the follow-up survey), the study authors note that “sample size was originally set assuming 7 clusters per condition. 25 participants per cluster (185 total per condition) provided 80% power to detect a between-group different in HIV test of 16 percentage points or more. Fiscal constraints required us to scale back the number of clusters to 2.”

8 Serial cross-sectional study where the intervention was an online ad campaign delivered through four sites/access points; many potential confounders. Study authors assessed time as a confounder (seasonal differences in syphilis testing) by comparing the before-after in the campaign year to the two previous years, but did not report what other variables/confounders they considered.

9 Because the outcome was “number of syphilis tests requested” there was no predetermined denominator (number of participants). If individuals requested syphilis testing multiple times during the 7 week pre-campaign period or 7 week intervention period, they would have been double-counted.

10 The study authors used multivariable Poisson regression to calculate the rate ratio of syphilis testing frequency, post-period compared to pre-period, but provided no information on how they calculated rates from pure "number of tests in each period" data nor on what variables they used in their multivariable regression.
