# Supplementary material for: Web-Based Service Provision of HIV, Viral Hepatitis, and Sexually Transmitted Infection Prevention, Testing, Linkage, and Treatment for Key Populations: Systematic Review and Meta-analysis
Source: J Med Internet Res. 2022 Dec 22;24(12):e40150. doi: 10.2196/40150 (PMC9816952; doi:10.2196/40150)
Supplement: Multimedia Appendix 3 [file jmir_v24i12e40150_app3.pdf]

Appendix C. GRADE tables presenting summary of evidence used for the effectiveness review.

Online outreach

Certainty assessment							№ of patients		Effect		Certainty	Importance
№ of studies	Study design	Risk of bias	Inconsistency	Indirectness	Imprecision	Other considerations	outreach through online platforms	standard of care	Relative (95% CI)	Absolute (95% CI)		
**Previously unreached people getting reached (follow up: 6 months; assessed with: Number of contacts with MSM by public health dept)**												
1 [1,a]	observational studies	serious [b]	not serious [c]	not serious	not serious	none	When only traditional outreach methods were used (Oct 2011-Mar 2012), the local Public Health dept had contact with 60 MSM. After implementing outreach via Grindr (Oct 2013 - Mar 2014), the dept had contact with 816 MSM, a >1500% increase ($p<0.001$).				⊕⊕⊕◯ MODERATE [d]	CRITICAL
**Use of prevention services (follow up: range 3 months to 12 months; assessed with: condom use, self-reported)**												
1 [2,e]	randomised trials	not serious	not serious [c]	not serious	not serious	none		73.0%	**RR 1.00** (0.86 to 1.17)	**0 fewer per 1,000** (from 102 fewer to 124 more)	⊕⊕⊕⊕ HIGH [f]	CRITICAL
**Use of prevention services (follow up: mean 6 months; assessed with: consistent condom use, self-reported, with main male sex partner)**												
1 [3,g]	randomised trials	serious [h]	not serious [c]	not serious	serious [i]	none	15/37 (40.5%)	18/45 (40.0%)	**RR 0.90** (0.39 to 2.06)	**40 fewer per 1,000** (from 244 fewer to 424 more)	⊕⊕◯◯ LOW [j]	CRITICAL
**Use of prevention services (follow up: mean 6 months; assessed with: consistent condom use, self-reported, with casual or commercial male sex partner)**												
1 [3,g]	randomised trials	serious [h]	not serious [c]	not serious	serious [i]	none	14/25 (56.0%)	16/31 (51.6%)	**RR 0.95** (0.41 to 2.17)	**26 fewer per 1,000** (from 305 fewer to 604 more)	⊕⊕◯◯ LOW [k]	CRITICAL
**Use of prevention services (follow up: mean 6 months; assessed with: consistent condom use, self-reported, during receptive anal sex with male sex partner)**												

Certainty assessment							№ of patients		Effect		Certainty	Importance
№ of studies	Study design	Risk of bias	Inconsistency	Indirectness	Imprecision	Other considerations	outreach through online platforms	standard of care	Relative (95% CI)	Absolute (95% CI)		
1 [3,g]	randomised trials	serious [h]	not serious [c]	not serious	serious [i]	none	9/23 (39.1%)	15/29 (51.7%)	**RR 0.40** (0.12 to 1.32)	**310 fewer per 1,000** (from 455 fewer to 166 more)	⊕⊕◯◯ LOW [l]	CRITICAL
**Use of prevention services (follow up: mean 6 months; assessed with: consistent condom use, self-reported, during insertive anal sex with male sex partner)**												
1 [3,g]	randomised trials	serious [h]	not serious [c]	not serious	serious [i]	none	14/29 (48.3%)	15/31 (48.4%)	**RR 0.60** (0.23 to 1.54)	**194 fewer per 1,000** (from 373 fewer to 261 more)	⊕⊕◯◯ LOW [m]	CRITICAL
**Use of testing services (follow up: range 3 months to 6 months; assessed with: HIV testing)**												
2 [2,3,e,g]	randomised trials	serious [h]	not serious	not serious	not serious	none		34/50 (68.0%)	**RR 1.39** (1.21 to 1.60)	**265 more per 1,000** (from 143 more to 408 more)	⊕⊕⊕◯ MODERATE [n,o,p]	CRITICAL
								43.0%		**168 more per 1,000** (from 90 more to 258 more)		
**Use of testing services (follow up: range 3 months to 12 months; assessed with: Syphilis testing)**												
1 [2,e]	randomised trials	not serious	not serious [c]	not serious	serious [q]	none		5.0%	**RR 0.92** (0.70 to 1.21)	**4 fewer per 1,000** (from 15 fewer to 10 more)	⊕⊕⊕◯ MODERATE [f]	CRITICAL

CI: Confidence interval; **RR:** Risk ratio

Explanations

a. Study description: This serial cross-sectional study among MSM in the USA compared the number of MSM engaged in HIV/STI services through Grindr vs standard outreach methods.

b. Risk of bias (assessed through ROBINS-I): Potential bias due to confounding (two years separated the two data collection time periods, and through use of Grindr for outreach was the main intervention, the public health dept may have adjusted other methods of reaching out to potential HIV/STI service users). There was no denominator (population) to calculate rates; only number of contacts - which may have included duplicates. For the uptake of prevention/testing services outcome, no comparisons to pre-Grindr outreach were reported.

c. Inconsistency: This could not be evaluated, as there is only a single study.

d. Additional data: Non-comparative descriptive data after implementing Grindr outreach. Among the Grindr contacts, 68% remained engaged after the avatar identified as an outreach health educator. Of those contacts who remained engaged, 35% received some combination of counseling, referrals, testing, treatment, and/or followup. For engaged Grindr users who self-identified for testing encounters, 14 tested for HIV/gonorrhea/chlamydia (1 case of pharyngeal chlamydia, 2 cases of rectal chlamydia, 1 case of urogenital chlamydia, and 1 new HIV infection were identified) and 13 tested for syphilis (1 case of late latent syphilis was identified).

e. Study description: This stepped-wedge cluster RCT among 1381 MSM in China compared an integrated online HIV testing intervention (multimedia HIV testing campaign, online HIV testing service, and local testing promotion campaigns tailored for MSM) to conventional HIV testing programs routinely provided by local CDCs and CBOs.

f. Control (standard of care) risk based on baseline data from N=1318 participants in the Tang 2018 study.

g. Study description: This RCT among 100 MSM in China compared online HIV self-testing via WeTest (a private WeChat group which provided app-based messages and referrals to HIV services) and watching a brief video about self-administering the oral HIV self-test kit.

h. Risk of bias: Downgraded once for detection bias. Blinding was not possible given the nature of the intervention. Detection bias was possible as data was self-reported and may have been affected by a lack of blinding.

i. Imprecision: Downgraded because 95% CI for RR includes both 1 (no effect) AND either appreciable harm (0.75) or appreciable benefit (1.25). The study also had a small sample size (n<=50 in each arm) but we did not downgrade again for this.

j. Additional data: RR reported in GRADE table is the adjusted RR reported by study authors, accounting for group, time, age, education, income, occupation, and hukuo (Chinese household registration). We calculated the crude risk ratio (RR: 1.01, 95% CI: 0.60-1.72).

k. Additional data: RR reported in GRADE table is the adjusted RR reported by study authors, accounting for group, time, age, education, income, occupation, and hukuo (Chinese household registration). We calculated the crude risk ratio (RR: 1.09, 95% CI: 0.67-1.77).

l. Additional data: RR reported in GRADE table is the adjusted RR reported by study authors, accounting for group, time, age, education, income, occupation, and hukuo (Chinese household registration). We calculated the crude risk ratio (RR: 0.76, 95% CI: 0.41-1.41).

m. Additional data: RR reported in GRADE table is the adjusted RR reported by study authors, accounting for group, time, age, education, income, occupation, and hukuo (Chinese household registration). We calculated the crude risk ratio (RR: 1.00, 95% CI: 0.59-1.68).

n. Additional data: Tang et al. ran other analytical models (i.e. intervention effect assuming fixed secular trend, per protocol effect assuming fixed secular trend across clusters, intervention effect adjusted for province, intervention effect using multiple imputation) but all reported a relative risk between 1.43 and 1.49 and p<0.001. For meta-analysis, we chose to use the model adjusted for age, marital status and income. This study also reported the HIV testing in the past 3 months outcome disaggregated by age group (<=30 and >30 years old) and by in-person community activities (with and without).

o. Additional data: Zhu et al. also provided self-reported data for any oral HIV testing in the past 6 months (OR: 2.17, 95% CI: 1.08-4.37).

p. Control (standard of care) includes 1) events/participants from the Zhu 2019 study and 2) risk based on baseline data from N=1318 participants in the Tang 2018 study.

q. Imprecision: Downgraded because 95% CI for RR includes both 1 (no effect) AND either appreciable harm (0.75) or appreciable benefit (1.25).

References
1. Lampkin, D., Crawley, A., Lopez, T. P., Mejia, C. M., Yuen, W., Levy, V.. Reaching Suburban Men Who Have Sex With Men for STD and HIV Services Through Online Social Networking Outreach: A Public Health Approach. J Acquir Immune Defic Syndr; May 1 2016.

2. Tang, Weiming, Wei, Chongyi, Cao, Bolin, Wu, Dan, Li, Katherine T., Lu, Haidong, Ma, Wei, Kang, Dianmin, Li, Haochu, Liao, Meizhen, Mollan, Katie R., Hudgens, Michael G., Liu, Chuncheng, Huang, Wenting, Liu, Aifeng, Zhang, Ye, Smith, M. Kumi, Mitchell, Kate M., Ong, Jason J., Fu, Hongyun. Crowdsourcing to expand HIV testing among men who have sex with men in China: A closed cohort stepped wedge cluster randomized controlled trial. PLoS Med; 2018.

3. Zhu, X., Zhang, W., Operario, D., Zhao, Y., Shi, A., Zhang, Z., Gao, P., Perez, A., Wang, J., Zaller, N., Yang, C., Sun, Y., Zhang, H.. Effects of a Mobile Health Intervention to Promote HIV Self-testing with MSM in China: A Randomized Controlled Trial. AIDS Behav; Nov 2019.

Online case management

Certainty assessment							№ of patients		Effect		Certainty	Importance
№ of studies	Study design	Risk of bias	Inconsistency	Indirectness	Imprecision	Other considerations	online case management	standard of care	Relative (95% CI)	Absolute (95% CI)		
**Use of prevention services (follow up: 6 months; assessed with: PrEP adherence (TFV-DP DBS concentrations ≥700 fmol/punch, equivalent to ≥4 doses of TDF/week))**												
1 [1,a]	randomised trials	serious [b]	not serious [c]	not serious	serious [d]	none	36/73 (49.3%)	30/68 (44.1%)	**RR 1.12** (0.78 to 1.59)	**53 more per 1,000** (from 97 fewer to 260 more)	⨁⨁◯◯ LOW [e]	CRITICAL
**Uptake of testing services (follow up: 8 months; assessed with: Repeat HIV testing (self-reported >1 test vs 0/1 test between baseline and followup assessment))**												
1 [2,f]	randomised trials	very serious [g]	not serious [c]	not serious	serious [d]	none	22/44 (50.0%)	19/47 (40.4%)	**RR 1.24** (0.78 to 1.95)	**97 more per 1,000** (from 89 fewer to 384 more)	⨁◯◯◯ VERY LOW [h]	CRITICAL
**Treatment initiation (follow up: 90 days; assessed with: Linkage to care (completion of at least one HIV-related laboratory test (HIV viral load or CD4 count) within 90 days after release into the community))**												

Certainty assessment							№ of patients		Effect		Certainty	Importance
№ of studies	Study design	Risk of bias	Inconsistency	Indirectness	Imprecision	Other considerations	online case management	standard of care	Relative (95% CI)	Absolute (95% CI)		
1 [3,i]	observational studies	serious [j]	not serious [c]	not serious	serious [d]	none	107/144 (74.3%)	64/94 (68.1%)	**RR 1.09** (0.92 to 1.29)	**61 more per 1,000** (from 54 fewer to 197 more)	⊕⊕◯◯ LOW [k]	CRITICAL
**Treatment initiation (follow up: 6 months; assessed with: Received primary HIV care within the last 6 months (self-reported))**												
1 [4,l]	observational studies	serious [m]	not serious [c]	not serious	serious [n]	none	54/60 (90.0%)	45/60 (75.0%)	**RR 1.20** (1.01 to 1.42)	**150 more per 1,000** (from 8 more to 315 more)	⊕⊕◯◯ LOW	CRITICAL
**Treatment retention/completion (follow up: range 4 months to 6 months; assessed with: ART adherence in the past 30 days)**												
1 [5,o]	randomised trials	serious [p]	not serious [c]	not serious	not serious	none	At the four-month (end of active intervention) timepoint, higher overall ART adherence in intervention than control (89.0% (95% CI: 83.4-94.6) intervention vs 77.2% (95% CI: 66.7-87.7) control, difference 11.8% (95% CI: 0.34-23.2), p=0.04). However, improvements in adherence were not sustained at the 6-month assessment (85.3% (95% CI: 80.0-90.6) intervention vs 89.0% (95% CI: 83.2-94.9) control, difference -3.7% (95% CI: -11.4-4.0), p=0.34).				⊕⊕⊕◯ MODERATE [q]	CRITICAL
**Treatment retention/completion (follow up: 6 months; assessed with: Engagement in HIV care (having seen an HIV care provider in the community at least once in the past 24 weeks))**												
1 [6,r]	randomised trials	serious [b]	not serious [c]	not serious	serious [n]	none	44/50 (88.0%)	45/50 (90.0%)	**RR 0.98** (0.85 to 1.12)	**18 fewer per 1,000** (from 135 fewer to 108 more)	⊕⊕◯◯ LOW [s]	CRITICAL
**Treatment retention/completion (follow up: 6 months; assessed with: Currently taking ART (self-reported))**												

Certainty assessment							№ of patients		Effect		Certainty	Importance
№ of studies	Study design	Risk of bias	Inconsistency	Indirectness	Imprecision	Other considerations	online case management	standard of care	Relative (95% CI)	Absolute (95% CI)		
1 [4,l]	observational studies	serious [m]	not serious [c]	not serious	serious [d]	none	50/60 (83.3%)	42/60 (70.0%)	**RR 1.19** (0.97 to 1.45)	**133 more per 1,000** (from 21 fewer to 315 more)	⨁⨁◯◯ LOW	CRITICAL
**Viral load (follow up: 6 months; assessed with: Viral suppression (viral load < 200 copies/ml))**												
1 [6,r]	randomised trials	serious [b]	not serious [c]	not serious	serious [d]	none	28/50 (56.0%)	29/50 (58.0%)	**RR 0.97** (0.69 to 1.36)	**17 fewer per 1,000** (from 180 fewer to 209 more)	⨁⨁◯◯ LOW [t]	CRITICAL
**Viral load (assessed with: complete virologic suppression at any of the first 6 visits)**												
1 [7,u]	observational studies	serious [v]	not serious [c]	not serious	not serious	none	468/514 (91.1%)	408/687 (59.4%)	**RR 1.53** (1.43 to 1.64)	**315 more per 1,000** (from 255 more to 380 more)	⨁⨁⨁◯ MODERATE [w]	CRITICAL
**Viral load (follow up: 6 months; assessed with: Viral suppression (viral load < 200 copies/ml, self-reported))**												
1 [4,l]	observational studies	serious [m]	not serious [c]	not serious	serious [d]	none	37/54 (68.5%)	28/43 (65.1%)	**RR 1.05** (0.79 to 1.40)	**33 more per 1,000** (from 137 fewer to 260 more)	⨁⨁◯◯ LOW	CRITICAL

CI: Confidence interval; **RR:** Risk ratio

Explanations

a. Study description: This RCT among 200 HIV- 15-19yo MSM and TG women in Thailand compared using a mobile app with youth friendly services AND PrEP self-assessment, rewards, and appointment reminders with a mobile app with youth friendly services only.

b. Risk of bias: Downgraded once because some concerns of bias. Details of the randomisation and/or allocation process are not documented. No additional information on allocation concealment; some baseline differences between groups in terms of sociodemographics and outcome measures at baseline suggest potential issues.

c. Inconsistency: This could not be evaluated, as there is only a single study.

d. Imprecision: Downgraded once because 95% CI for RR includes both 1 (no effect) AND either appreciable harm (0.75) or appreciable benefit (1.25). Small sample size in both arms (but did not downgrade again).

e. Additional data: Songtaweesin et al. reported aOR: 1.18 (95% CI: 0.58-2.42) adjusted for gender identity, age, number of sex partners, and self-perceived risk for HIV infection. Authors also presented data for the 3mo timepoint: 44/81 (54.3%) intervention vs 40/79 (50.6%) control, RR 1.07 (95% CI: 0.80-1.44), aOR 1.08 (95% CI: 0.54-2.16).

f. Study description: This RCT among 113 HIV- MSM in the USA compared using a mobile app (Status Update Project) with a monthly My Health Survey to recommend next HIV test date, prevention 411 with HIV/STI information, etc to no treatment.

g. Risk of bias: Downgraded twice because of high risk of bias (some concerns across multiple domains). Details of the randomisation and/or allocation process are not documented. No additional information on allocation concealment; some baseline differences between groups in terms of sociodemographics and outcome measures at baseline suggest potential issues. By 8-month follow-up, loss-to-follow-up rate was 21% in the intervention arm and 12% in the control arm; differences in missingness could depend on the true value but we judged it unlikely. Given the intervention of interest (online case mgmt.), blinding was not possible for participants and personnel. Deviations from the intended intervention due to lack of blinding were not documented. We judged that self-reported outcomes (with no validation from lab/other measures) were potentially influenced by lack of blinding (potential detection bias).

h. Additional data: Horvath et al. reported data at the 4month followup assessment as well: 8/47 (17.0%) intervention vs 2/52 (3.8%) control, RR: 4.4 (95% CI: 0.9-19.8), aRR 3.4 (95% CI: 0.7-15.6) adjusting for demographic, behavioral, and HIV testing intention and behavior variables.

i. Study description: This cohort study among 238 HIV+ adults soon to be released from prison in the USA compared an online tailored personalized website for HIV/STI testing to access to an online provider directory webpage only.

j. Risk of bias: Cohort study assessed with ROBINS-I. Baseline and time-varying confounding was not adjusted for by the variables available in the study. Selection of participants into the study was not based on participant characteristics observed after the start of the intervention, but the start of follow-up and start of intervention did not coincide for most participants (intervention group was those who successfully received the case management video conference in time for their release from prison; control group was those who did not receive the video conference in time).

k. Additional data: Brantley et al. reported aOR 1.2 (95% CI: 0.6-2.3), adjusting for race, birth sex, age, HIV transmission risk, time since HIV diagnosis, AIDS diagnosis history, baseline (pre-release) viral suppression status, HIV diagnosis status prior to incarceration, and HIV care engagement status prior to incarceration.

l. Study description: This cohort study among 120 HIV+ 18-34yo MSM and TG women in the USA compared those who completed a 6-month digital HIV care navigation (text messaging with personal HIV care navigator) to those who did not complete the 6 month intervention.

m. Risk of bias: Cohort study assessed using ROBINS-I. Baseline and time-varying confounding was not adjusted for by the variables available in the study. Among the 120 participants enrolled in the intervention, 60 were lost to follow up and did not complete the intervention. The 60 who completed the intervention were considered the intervention group, and the 60 who were lost to follow up (67% for unknown reasons, 10% for phone loss, 15% for moving out of jurisdiction, and other reasons) were considered the control group. However, characteristics of the control group were not different from the overall sample (or the intervention group). Given the intervention of interest (online case mgmt.), blinding was not possible for participants and personnel and outcome was self-reported.

n. Imprecision: Downgraded once because small sample size in both arms.

o. Study description: This RCT among 90 HIV+ stimulant using MSM in the USA compared using a mobile app (APP+) with IMB HIV/ART content, a choose your own adventure story, and medication self-monitoring to no treatment.

p. Risk of bias: Given the intervention of interest (online case mgmt.), blinding was not possible for participants and personnel. However, deviations from the intended intervention due to lack of blinding were not documented, and any potential deviations were unlikely to have affected the outcome. We judged that self-reported outcomes (with no validation from lab/other measures) were potentially influenced by lack of blinding (detection bias).

q. Additional data: Horvath et al. reported no significant differences at any assessment timepoints between intervention and control for other measurements of ART adherence: taking ART within 2 hours of the scheduled time, adherence while using stimulants, taking ART within 2 hours of the scheduled time while using stimulants, almost always or always (vs never, rarely, sometimes, or usually) taking ART doses correctly in the past 30 days.

r. Study description: This RCT among 110 HIV+ adults soon to be or recently released from prison in the USA compared the CARE+Corrections intervention (computerized motivational interview and individual risk reduction plan pre-release plus text messaging about care navigation post-release) to an attention-control (opioid overdose prevention video and HIV providers/resources printout).

s. Additional data: Kuo et al. present aOR 1.18 (95% CI: 0.25-5.53), adjusting for the location of study enrollment, gender, sexual orientation, depressive symptomatology, and positive PTSD score.

t. Additional data: Kuo et al. present aOR 2.04 (95% CI: 0.62-6.70), adjusting for the location of study enrollment, gender, sexual orientation, depressive symptomatology, and positive PTSD score.

u. Study description: This cohort study among 1201 HIV+ prisoners in the USA compared those using telemedicine for HIV care to on-site management by correctional facility physicians.

v. Risk of bias: Cohort study assessed with ROBINS-I. Downgraded once for moderate concerns in one risk of bias domain. Baseline and time-varying confounding was not adjusted for by the variables available in the study. Control group was prisoners who received HIV care via standard of care on-site management by physicians before the prison implemented telemedicine / online case management.

w. Additional data: Young et al. report: The proportion of subjects with complete virologic suppression during the first 6 visits was significantly greater in the telemedicine group (91.1% vs 59.3%; OR, 7.0 [95% CI, 5.1–9.8]; P < .001), even when removing all subjects who were suppressed at the first visit (75.8% vs 23.0%; OR, 10.5 [95% CI, 6.9–16.1]; P < .001), and controlling for the total number of clinic visits in a logistic regression analysis (OR, 4.2 [95% CI, 2.5–7.0]; P < .001).

References

1. Songtaweesin, W. N., Kawichai, S., Phanuphak, N., Cressey, T. R., Wongharn, P., Saisaengjan, C., Chinbunchorn, T., Janyam, S., Linjongrat, D., Puthanakit, T.. Youth-friendly services and a mobile phone application to promote adherence to pre-exposure prophylaxis among adolescent men who have sex with men and transgender women at-risk for HIV in Thailand: a randomized control trial. J Int AIDS Soc; Sep 2020.

2. Horvath, Keith J., Lammert, Sara, Danh, Thu, Mitchell, Jason W.. The feasibility, acceptability and preliminary impact of mobile application to increase repeat hiv testing among sexual minority men. AIDS Behav; 2019.

3. Brantley, A. D., Page, K. M., Zack, B., Friedrich, K. R., Wendell, D., Robinson, W. T., Gruber, D.. Making the Connection: Using Videoconferencing to Increase Linkage to Care for Incarcerated Persons Living with HIV Post-release. AIDS Behav; 2019.

4. Arayasirikul, S., Turner, C., Trujillo, D., Le, V., Wilson, E. C.. Efficacy and Impact of Digital HIV Care Navigation in Young People Living With HIV in San Francisco, California: Prospective Study. JMIR Mhealth Uhealth; 2020.

5. Horvath, Keith J., Lammert, Sara, MacLehose, Richard F., Danh, Thu, Baker, Jason V., Carrico, Adam W.. A pilot study of a mobile app to support HIV antiretroviral therapy adherence among men who have sex with men who use stimulants. AIDS Behav; 2019.

6. Kuo, I., Liu, T., Patrick, R., Trezza, C., Bazerman, L., Uhrig Castonguay, B. J., Peterson, J., Kurth, A., Beckwith, C. G.. Use of an mHealth Intervention to Improve Engagement in HIV Community-Based Care Among Persons Recently Released from a Correctional Facility in Washington, DC: A Pilot Study. AIDS Behav; 2019.

7. Young, Jeremy D., Patel, Mahesh, Badowski, Melissa, Mackesy-Amiti, Mary Ellen, Vaughn, Pyrai, Shicker, Louis, Puisis, Michael, Ouellet, Lawrence J.. Improved virologic suppression with HIV subspecialty care in a large prison system using telemedicine: an observational study with historical controls. Clin Infect Dis; 2014.

Targeted online health information

Certainty assessment							№ of patients		Effect		Certainty	Importance
**№ of studies**	**Study design**	**Risk of bias**	**Inconsistency**	**Indirectness**	**Imprecision**	**Other considerations**	**targeted online health information**	**standard of care**	**Relative (95% CI)**	**Absolute (95% CI)**		
**Use of prevention services (follow up: 30 days; assessed with: Vaccination for hepatitis A or B, HPV, or meningococcal meningitis)**												
1 [1,a]	randomised trials	serious [b]	not serious [c]	serious [d]	serious [e]	none	0/68 (0.0%)	0/36 (0.0%)	not estimable		⊕◯◯◯ VERY LOW	CRITICAL
**Uptake of testing services for HIV/VH/STIs (follow up: 12 weeks; assessed with: Followed up for HIV test result (after requesting and returning HIV test kit))**												
1 [2,f]	randomised trials	not serious	not serious [c]	serious [g]	serious [h]	none	8/57 (14.0%)	0/55 (0.9%)	**RR 3.56** (0.32 to 39.65)	**23 more per 1,000** (from 6 fewer to 351 more)	⊕⊕◯◯ LOW [i]	CRITICAL
**Uptake of testing services for HIV/VH/STIs (follow up: 30 days; assessed with: Tested for HIV or STIs)**												
1 [1,a]	randomised trials	serious [b]	not serious [c]	serious [d]	serious [j]	none	22/68 (32.4%)	8/36 (22.2%)	**RR 1.46** (0.72 to 2.94)	**102 more per 1,000** (from 62 fewer to 431 more)	⊕◯◯◯ VERY LOW	CRITICAL
**Uptake of testing services for HIV/VH/STIs (follow up: 30 days; assessed with: HIV testing among those who tested for any HIV/STIs)**												

Certainty assessment							№ of patients		Effect		Certainty	Importance
№ of studies	Study design	Risk of bias	Inconsistency	Indirectness	Imprecision	Other considerations	targeted online health information	standard of care	Relative (95% CI)	Absolute (95% CI)		
1 [1,a]	randomised trials	very serious [k]	not serious [c]	serious [d]	very serious [l]	none	18/22 (81.8%)	6/8 (75.0%)	**RR 1.09** (0.70 to 1.70)	**68 more per 1,000** (from 225 fewer to 525 more)	⨁◯◯◯ VERY LOW	CRITICAL
**Uptake of testing services for HIV/VH/STIs (follow up: 30 days; assessed with: Gonorrhea testing among those who tested for any HIV/STIs)**												
1 [1,a]	randomised trials	very serious [k]	not serious [c]	serious [d]	very serious [l]	none	9/22 (40.9%)	2/8 (25.0%)	**RR 1.64** (0.45 to 6.01)	**160 more per 1,000** (from 138 fewer to 1,000 more)	⨁◯◯◯ VERY LOW	CRITICAL
**Uptake of testing services for HIV/VH/STIs (follow up: 30 days; assessed with: Chlamydia testing among those who tested for any HIV/STIs)**												
1 [1,a]	randomised trials	very serious [k]	not serious [c]	serious [d]	very serious [l]	none	12/22 (54.5%)	2/8 (25.0%)	**RR 2.18** (0.62 to 7.69)	**295 more per 1,000** (from 95 fewer to 1,000 more)	⨁◯◯◯ VERY LOW	CRITICAL
**Uptake of testing services for HIV/VH/STIs (follow up: 30 days; assessed with: Syphilis testing among those who tested for any HIV/STIs)**												
1 [1,a]	randomised trials	very serious [k]	not serious [c]	serious [d]	very serious [l]	none	14/22 (63.6%)	2/8 (25.0%)	**RR 2.55** (0.74 to 8.81)	**387 more per 1,000** (from 65 fewer to 1,000 more)	⨁◯◯◯ VERY LOW	CRITICAL
**Uptake of testing services for HIV/VH/STIs (follow up: 30 days; assessed with: Anal pap smear among those who tested for any HIV/STIs)**												
1 [1,a]	randomised trials	very serious [k]	not serious [c]	serious [d]	very serious [l]	none	1/22 (4.5%)	1/8 (12.5%)	**RR 0.36** (0.03 to 5.15)	**80 fewer per 1,000** (from 121 fewer to 519 more)	⨁◯◯◯ VERY LOW	CRITICAL

Certainty assessment							№ of patients		Effect		Certainty	Importance
№ of studies	Study design	Risk of bias	Inconsistency	Indirectness	Imprecision	Other considerations	targeted online health information	standard of care	Relative (95% CI)	Absolute (95% CI)		
**Use of testing services for HIV/VH/STIs (follow up: 7 weeks; assessed with: Syphilis testing)**												
1 [3,m]	observational studies	serious [n]	not serious [c]	serious [o]	not serious	none	2025/-	2049/-	**Rate ratio 1.00** (0.94 to 1.07)	**-- per 1000 patient(s) per years** (from -- to --)	⨁⨁◯◯ LOW	CRITICAL

CI: Confidence interval; **RR:** Risk ratio

Explanations

a. Study description: This RCT among 130 MSM in USA compared a tailored website (including content customized to the user based on prior testing experiences and motivations, barriers and resources to testing, and important values - information gathered about each study participant during baseline measurement) to access to an online provider directory with no tailored content. N=86 were allocated to the intervention arm and n=44 to the control.

b. Risk of bias: Downgraded once for detection bias. Blinding was not possible given the nature of the intervention. Detection bias was possible as data was self-reported and may have been affected by a lack of blinding. Also, no information on randomization/allocation methods was reported (appears to be 2:1 allocation to the intervention:control).

c. Inconsistency: This could not be evaluated, as there is only a single study.

d. Indirectness: Downgraded once because intervention was not exactly "targeted health information." Intervention was an online health intervention (Get Connected! website) tailored to study participants based on sociodemographic/behavioral/motivational information, but this intervention was not exactly "targeted health information" per se because the information used for tailoring was collected through the study's baseline assessment.

e. Imprecision: Downgraded because no events and low sample size in both arms.

f. Study description: This RCT among 112 MSM in USA compared using social networks/peer leaders on Facebook delivering HIV information in group settings and individually (via chat, wall posts, and personal messages) to peer leaders on Facebook delivering general health information.

g. Indirectness: Downgraded once because intervention was not exactly "targeted health information." Intervention was a social networking intervention with peer leaders on facebook in study-created groups of 12 to disseminate health information to their group members through sending chat messages and wall posts (general conversation and HIV prevention/testing information, tailored to participant response/engagement), not exactly "targeted health information".

h. Imprecision: Downgraded because no event in control arm and low sample size in both arms. RR calculated using continuity correction of 0.5; study authors reported number of events and percentages per arm only, with no statistical analyses.

i. Additional data: The study also reports outcomes that are precursors to the outcome we present in the GRADE table (followed up to receive HIV test result). The study reported that 25/57 (44%) in the intervention arm requested a HIV test kit, compared to 11/55 (20%) in the control arm (RR: 2.19, 95% CI: 1.20-4.01). Of those who requested a HIV test kit, 9 in the intervention arm returned the HIV test kit (9/25, 36%) compared to 2 in the control arm (2/11, 18%) (RR: 1.98, 95% CI: 0.51-7.70).

j. Imprecision: Downgraded because 95% CI for RR includes both 1 (no effect) AND either appreciable harm (0.75) or appreciable benefit (1.25). The study also had a small sample size (n<=50 in each arm) but we did not downgrade again for this.

k. Risk of bias: Downgraded twice: 1) for detection bias, as blinding was not possible given the nature of the intervention. Detection bias was possible as data was self-reported and may have been affected by a lack of blinding. 2) because this was a sub-group of the "uptake of testing for HIV/STIs" above so the intervention and control groups were not randomized per se.

l. Imprecision: Downgraded twice, 1) because 95% CI for RR includes both 1 (no effect) AND either appreciable harm (0.75) or appreciable benefit (1.25), and 2) this is a subgroup of the row above so a very small sample size (n=22 in the intervention arm and n=8 in the control).

m. Study description: This serial cross-sectional study among MSM in Canada compared the number of syphilis tests ordered (public health laboratory data) in the 7 weeks prior to the launch of the ad campaign to the number of tests ordered in the 7 weeks after the first ads appeared.

n. Risk of bias: The study authors used multivariable Poisson regression to calculate the rate ratio of syphilis testing frequency, post-period compared to pre-period, but provided no information on how they calculated rates from pure "number of tests in each period" data nor on what variables they used in their multivariable regression.

o. Indirectness: Downgraded once because the intervention was not exactly "targeted health information". Intervention was a social media syphilis testing campaign, where ads were hosted on four online media platforms: Grindr, Facebook, Squirt and the Gay Ad Network. When clicked, ads would direct the user to an information website on the syphilis outbreak and the importance of testing. Ads were targeted in that they were geo-tagged for location and the specific media platforms had specific target populations (clients) but otherwise not exactly "targeted health information".

References

1. Bauermeister, J. A., Pingel, E. S., Jadwin-Cakmak, L., Harper, G. W., Horvath, K., Weiss, G., Dittus, P.. Acceptability and preliminary efficacy of a tailored online HIV/STI testing intervention for young men who have sex with men: the Get Connected! program. AIDS Behav; Oct 2015.

2. Young, S. D., Cumberland, W. G., Lee, S. J., Jaganath, D., Szekeres, G., Coates, T.. Social networking technologies as an emerging tool for HIV prevention: a cluster randomized trial. Ann Intern Med; Sep 3 2013.

3. Ross, C., Shaw, S., Marshall, S., Stephen, S., Bailey, K., Cole, R., Wylie, J., Bullard, J., Van Caeseele, P., Reimer, J., Plourde, P.. Impact of a social media campaign targeting men who have sex with men during an outbreak of syphilis in Winnipeg, Canada. Can Commun Dis Rep; Feb 4 2016.
