# Supplementary material for: Web-Based Service Provision of HIV, Viral Hepatitis, and Sexually Transmitted Infection Prevention, Testing, Linkage, and Treatment for Key Populations: Systematic Review and Meta-analysis
Source: J Med Internet Res. 2022 Dec 22;24(12):e40150. doi: 10.2196/40150 (PMC9816952; doi:10.2196/40150)
Supplement: Multimedia Appendix 4 [file jmir_v24i12e40150_app4.pdf]

Appendix D. Description of and key findings from studies included in the values and preferences review.

Online outreach

Study<br>Location<br>Population and sample size (n)	Study design and methods	Key values and preferences findings
Alarcón Gutiérrez et al., 2018<br><br>Spain: Barcelona<br><br>N=2656 MSM	Cross-sectional study	Unsolicited messages about rapid HIV, syphilis and hepatitis C testing were sent on apps for sexual and social encounters. A 38.4% response rate was obtained, 83.0% of them found it acceptable to receive the unsolicited message, and 73.2% effectiveness was obtained<br><br>Of the 2656 users (1029 in Grindr, 768 in PlanetRomeo and 859 in Wapo) contacted by the investigators, 1019 responded to the message and 846 responded favourably. 108 (12.8%) were interested in attending the programme facilities, 258 (30.5%) stated they were going to take the tests elsewhere or had already taken them, and the remaining 480 (56.7%) provided no information. Among the 108 users who were interested in attending the facilities, 79 attended, which gives an effectiveness of 73.2% without differences between apps (P=0.920).<br><br>The satisfaction survey, taken by 70 users (90.9%), presented an average score of 9.7 out of 10. All individuals (100%) who took this survey stated they would recommend this service to friends and acquaintances.
Cao et al., 2018<br><br>China<br><br>N=503 MSM	Cross-sectional study	MSM reported high interest (465/503, 92.4%) and willingness (463/503, 92.0%) to use a MSM-friendly physician finder function within gay mobile apps (which they also generally regarded as high in trustworthiness and were the second most commonly used platform to obtain information on STIs after search engines).
Ybarra et al., 2020<br><br>Uganda, Tanzania, Rwanda, South Sudan, and Kenya<br><br>N=2451 sexual and gender minority persons (self-identified LGBT)	Cross-sectional survey (recruited through Facebook ads and text messages on mobile carriers)	47.9% of respondents were "very likely" to engage in a sexual health programme for LGBT people if the outreach for the service was offered online and 41.1% if over text message or 43.1% over email<br>Over 50% of those recruited through social media said they would be very likely to engage with a sexual health program online or via email compared to about one in three in the mobile carrier recruited sample; when sex, sexual behaviour, and gender and sexual minority identities were held constant, the relative odds of being very likely to access web- and email-based interventions were significantly lower for those recruited through mobile carriers as compared to social media outreach efforts. However, those who were recruited through mobile carriers were no more likely to say they would engage in a text messaging-based program than those recruited through social media.
Brennan et al., 2018 and<br>Fantus et al., 2017<br><br>Canada: Ontario<br><br>N=18 frontline outreach workers and 2 managers	Qualitative: key informant interviews	Online technologies have reshaped the "gay/queer community", changed norms for social/sexual interactions, and can help reach out to hard-to-reach MSM:<br>• '"This is where people meet now. It's the new community. There's no gay community, physically, out here anymore. So it makes sense for organizations to step up and be where everybody else is" (Outreach worker; Toronto).<br>• "That's the type of model that we work on. We basically recruit people who are online who understand the technology world, understand the smartphone world, understand the now world. The way we cruise now is very different than the way we cruised 10 years ago" (Outreach worker; Ontario)<br>• "Online outreach is very crucial to those people who are not getting information, are not connecting to the right community that they need or where to get tested or just navigating coming out" (Outreach worker; Ontario).

from AIDS service organizations/CBOs, 2 public health volunteers serving MSM/TG		- We have really become able to disseminate so much information. So guys who are maybe more isolated and don't know what's going on in the community can use it as perhaps an access point to that information. Which I think is really important, especially so many of the men are older like 40 and up on [app]. So there are definitely some strength there and because the services never existed we get a lot of positive feedback and thank you's" (Outreach worker; Toronto). Online outreach is more non-intrusive and anonymous, yet also responsive to user needs: ''I never initiate conversations. It's one of the policies, it is passive outreach because with a lot of the apps and the sites, it's all based on sort of the consent of the community for you to be there. So I'll post an ad that says, 'hey, do you have questions about hooking up, oral sex, STI transmission, barebacking''' (Outreach worker; Ontario). ''I would definitely say the best part is the anonymity part. Another good thing about the online outreach that we do is the fact that it can be very instant, real time accessing a person'' (Outreach worker; Ontario). Online outreach also has some barriers, like quality of service, collaborations between outreach service agencies and companies that own apps and websites, budgetary and staff/volunteer capacity constraints, and data security/safety: ''I think one of the challenges is going to be consistent messaging, and from a quality control standpoint, that everyone is providing the same message, the same way.'' (Outreach worker; outside Ontario). ''One of the biggest fears that I might have, is that we really rely on the service providers and the site administrator, to allow us to be there. There are certain rules on each site and I know that over time, volunteers have mistakenly broken those rules. We've heard from the site administrators 'watch out or we can take away your membership'. So that's a vulnerability'' (Outreach Worker; Ontario). <br>Emerging ethical dilemmas and strategies to mitigate risk related to online outreach (four major themes): - managing personal and professional boundaries with clients: "It's hard to define between personal life and work life when it comes to outreach because it's going to be part of your natural conversation if you want to make friends; it's hard to describe the boundaries. You want to have fun, but you've got to work too. So, it's just like, ah." (Outreach worker; GTA) "You start chatting with a guy and something happen - there's always a possibility that there will be a connection. You have to make it very clear to the person you're communicating with that you're online in your role as either an outreach worker or an outreach volunteer." (Outreach worker; Ontario) - disclosing personal or identifiable information to clients: "When it comes to the big demographic stuff, age and sexual orientation and stuff, I have put that as being real; where I've had to be really, really careful, though, is to say what are you open to? I leave most of it open; I didn't check off that I wasn't into barebacking. So, somebody came back and said, oh, so you bareback. So I've got to be really careful about that sort of thing." (Outreach worker; Ontario) - maintaining client confidentiality and anonymity: "We struggle as an agency just trying to figure out how to use text messages in a way that you can ensure that the person receiving the text message is in fact the person you intended to receive it." (Manager; Ontario). - security and data storage measures of online information: "We are just deleting the messages. We're not keeping any record of them; we don't keep user names, and we're not printing chat transcripts…or reporting user names or IP addresses" (Outreach worker; Ontario).

Online case management

**Study**<br>**Location**	**Study design and methods**	**Key values and preferences findings**

Population and sample size (n)		
Balán et al., 2019<br><br>USA: New York City<br><br>N=59 mostly MSM	Qualitative: pilot test feedback	Participants "strongly favoured" having an online case management smartphone app for HIV/syphilis self- and partner-testing, interpretation of results, and linkage to care, but mentioned areas for improvement (e.g. having a disclaimer about the potential for receiving false positive results, logic flow of what information was on what tab within the app, and options for having a "guest" mode if testing for someone else whose data should not be saved on your smartphone)
Fontenot et al., 2019<br><br>USA: New York, Pennsylvania, Massachusetts<br><br>N=48 young MSM	Qualitative: online focus groups	Participants gave feedback on a web-enabled mobile app to provide HPV/vaccine information, referral, and linkage to care, noting that they preferred to Google things (or had too many other apps so were unlikely to use this single-purpose one), enjoyed using health apps for meditation and fitness but not certain about confidentiality for personal information
Fuchs et al., 2018<br><br>USA: Chicago, San Francisco<br><br>N=14 MSM	Qualitative: focus groups and in-depth interviews embedded in a larger RCT	Participants gave feedback on iText, a bidirectional weekly check-in messaging algorithm to support PrEP adherence, some stating that they found the app not helpful if they already "take the pill every morning religiously" but others stating that "getting those messages made me feel like there was always somebody there just in case something went wrong" or that it made them feel supported
Horvath et al., 2019b<br><br>USA: Miami, Orlando, Washington DC, Charlotte, Houston, New Orleans, San Francisco, Minneapolis<br><br>N=90 MSM (HIV+ stimulant users)	Qualitative: RCT pilot test feedback	APP+ (ART adherence tool) likes, frequency (illustrative quotes):<br>• Ease of use/user friendly, 25 ("Easy to navigate", "ease to log in", "user friendly", "simplicity of the layout"")<br>• Daily reminders, 19 ("notification remains until you take your meds, unlike an alarm that can be turned off and forgotten", "somehow having to record if I took my dose daily reminded me to take it daily")<br>• Acceptance and support, 17 ("It was very comforting and accepting in regards to my HIV", "motivational comments after logging")<br>• Weekly tracker, 10 ("gave me a percentage of my meds each week", "tool to use with my doctor", "ability to log data prior to the present day")<br>APP+ dislikes, frequency (illustrative quotes):<br>• App malfunctions, 15 ("app must remain open in order to function instead of running in the background", crashes, missed notifications)<br>• Daily reminders, 11 ("my alarm never goes off to alert me to take the meds", "setting the alarms was cumbersome", "the reminders were not well disguised so someone could see them on your notifications")<br>• General negative opinions, 9 ("easily done by using phone functions", "redundant questions", "time consuming")
Liu et al., 2020<br><br>USA: San Francisco, Atlanta<br><br>N=20 young MSM	Qualitative: pilot test feedback	Participants using DOT diary, a mobile app using AI and an electronic sexual diary to measure and support PrEP adherence, thought it highly acceptable, with median System Usability Scale scores in the "excellent" range.<br>Participants liked messaging that let them know how to get back to full protection (colour coding was a helpful signal), as well as daily reminders and a weekly calendar to track adherence/keep accountable/establish a routine (though some wanted to snooze reminders for later).
Refugio et al., 2018	Qualitative: pilot test feedback	Participants gave feedback on their experiences with PrEPTECH:<br>• 85% agreed that PrEPTECH is a better way for MSM to get PrEP at 90 and 180 days<br>• > 88% reported that PrEPTECH was very or extremely easy to use at 90 and 180 days

USA: San Francisco Bay Area<br><br>N=25 MSM (HIV-)		• All participants reported that PrEPTECH was very or extremely fast and convenient compared with other forms of getting on PrEP at 90 and 180 days.<br>• > 75% felt that PrEPTECH was very or extremely confidential and agreed that they would still use PrEPTECH even if the services were not free at 90 and 180 days.
Ventuneac et al., 2018<br><br>USA<br><br>N=495 MSM	Cross-sectional study	Most GBMSM (91%) reported interest in one or more sexual health features in apps. Features/functions in order of interest:<br>• find LGBT-friendly providers (83%)<br>• receive lab results (68%)<br>• schedule appointment reminders (67%)<br>• live chat with a healthcare provider (59%)<br>• receive medication reminder alerts (42%)<br>• track and receive feedback on their sexual behaviour (35%)<br>• track and receive feedback on their alcohol and drug use patterns (24%)<br>When ranking on a Likert scale, participants showed modest interest in integrating such features into existing apps already used was modest (M = 2.42, SD = 0.94); however 84% were at least “somewhat interested” in integrating these sexual health app features into existing mobile applications.

Targeted online health information

<table>
<tr><th>Study
Location
Population and sample size (n)</th><th>Study design and methods</th><th>Key values and preferences findings</th></tr>
<tr><td>Ross et al., 2016

Canada

N=NR MSM</td><td>Serial cross-sectional

Preferences were assessed by clicks to a syphilis testing website after launching a syphilis advertisement campaign on Facebook, the Gay Ad Network, Grindr, and Squirt</td><td>Syphilis testing campaign ads (~800,000) released over one month over four platforms elicited 2,166 clicks to the information website.
The Squirt eblast was the most effective at generating clicks per thousand appearances, but Grindr produced the greatest raw number of clicks (Grindr ads were clicked 1840 times, or 85% of all clicks, though Grindr only hosted 50% of all ad appearances), compared with Facebook and the Gay Ad Network, suggesting that “hookup” platforms were the most acceptable.
<table>
<tr><th>Platform</th><th>Appearances purchased</th><th>Clicks</th><th>Calculated clicks per thousand appearances</th></tr>
<tr><td>Facebook square ads</td><td>101,410</td><td>22</td><td>0.22</td></tr>
<tr><td>Gay Ad Network banners</td><td>101,520</td><td>25</td><td>0.25</td></tr>
<tr><td>Squirt eblast</td><td>4,550</td><td>91</td><td>20.00</td></tr>
<tr><td>Squirt banners</td><td>200,798</td><td>188</td><td>0.94</td></tr>
<tr><td>Grindr banners</td><td>~400,000</td><td>1,840</td><td>4.60</td></tr>
<tr><td>TOTAL</td><td>808,278</td><td>2,166</td><td></td></tr>
</table>
</td></tr>
<tr><td>Contesse et al., 2020

USA</td><td>Cross-sectional

Acceptability of HIV/STI partner notification using geospatial networking apps was assessed</td><td>Partner notification: greatest preference for (70%) and comfort with (77%) partners sharing through app, but less so for health department profiles (20% and 57% respectively) and anonymous messages in the app (10% and 41% respectively); though 50% preferred to notify a partner through their own profile, 26% preferred health department assistance and 24% an in-app anonymous messaging system</td></tr>
</table>

N=791 MSM	through ratings and rankings of notification methods in two hypothetical scenarios. Acceptability of health department presence on social apps was assessed through rating comfort with health departments having profiles on apps for providing information and services, what features of a department profile would make them more likely to think that it was real,<br>and how having health department profiles on an app would affect<br>their likelihood of using the app.	Preventive services: 82% were comfortable with apps allowing health department profiles to provide users with preventive sexual health services, with participants more or as likely to continue using the app if health departments had profiles on the app. Participants were more likely to consider a health department profile real if contact information for staff was provided, the app verified the profile as real, or the profile picture included an official health department logo
Kesten et al., 2019<br><br>England<br><br>N=25 MSM	Qualitative (semi-structured interviews)<br><br>Interviews explored sexual health information sources, perceptions and uses. Attitudes towards sexual health promotion through social media and dating apps were then discussed. The data were analysed using thematic analysis.	Most MSM positively responded to the idea of healthcare organizations providing sexual health information through social media/dating apps<br><br>Most MSM positively viewed timing of receiving information when browsing social media (time to absorb information discreetly)<br><br>Diverse acceptability by MSM, ranging from no impact to frustration to influencing careseeking/risk behaviors<br><br>Illustrative quotes:<br>• "When I saw the ‘get tested’ ad, well I thought it was great and it was, (…) but at the same time I feel a bit tracked, like why am I getting this on Instagram? It was okay because at the end I did the test and everything – it was a new thing that I found. I learned that it worked and it helped me but, at the same time, I couldn’t avoid feeling a bit tracked or targeted."<br>• "Mostly gay people (…) are not out, (…) and they have their family and friends and colleagues on Facebook. It’s the same with me, so I would not like it. If I liked the page, then they will be appearing on my page and so I would not prefer that."<br>• For me, personally, because (…) I feel like I’m on top of my sexual health, I feel like it’s [pop-up adverts] a little bit of an annoyance. It’s a little bit irritating because I’ve probably gone to try and hook up with a guy, not to think about having sexual health checkups, so I think it’s hard. I think they’re a good thing because they’re in a place where people are thinking about that, but at the same time (…), from a practical point of view, they are maybe a bit of an annoyance and they do get in the way. Sometimes they do have little banner ones [adverts] at the bottom I guess which are a bit more discrete; but quite often they’re quite in your face and that can be quite frustrating and probably I would suggest that if you’re spreading like sexual health messages then actually frustrating users is probably not an ideal way to do it.<br>• "Actually, when they [HIV campaign messages received on Grindr] first popped up saying ‘Know your status’ it reminded me that I hadn’t been tested in a while, so it actually prompted me to go and get a little blood test."

		• "I think it's a good idea [for healthcare organisations to use social media or dating apps to share sexual health information] and I think it's important to do so because by sharing that information from reputable sources people are more likely to trust the information that's been given to them and also people are more likely to say, 'oh, because I've had this information from these organisations, actually I'm more likely to get tested'."<br>• "I think it's the thing of not having to seek out that information, it's being given to people. It can be given in such an easy way to such a wide reach of people and it makes it a lot more accessible, especially for people who have access needs. It might not be easy for them to get to a clinic or speak to somebody about it, if they can see that out on social media it would make it a lot easier."<br>• "The HIV one was through GROWLr. It just said, If you answer these questions, you will be given a free HIV test." Now, I have no reason to have any thoughts that I would have HIV. I just thought That's a usefulthing to do."<br>• "Literally I need a pop up that comes up every five minutes just saying 'be safe'!"<br>• "It's such an easy way to specifically target gay men, or men-who-have-sex-with-men at least, and a lot of conversations on those apps will revolve around arranging sex and that kind of thing but to have the information there while you're also having those conversations makes it more – it puts it in your brain more prominently."
